# Modeling the TNFα-Induced Apoptosis Pathway in Hepatocytes

**DOI:** 10.1371/journal.pone.0018646

**Published:** 2011-04-20

**Authors:** Rebekka Schlatter, Kathrin Schmich, Anna Lutz, Judith Trefzger, Oliver Sawodny, Michael Ederer, Irmgard Merfort

**Affiliations:** 1 Institute for System Dynamics, University of Stuttgart, Stuttgart, Germany; 2 Department of Pharmaceutical Biology and Biotechnology, Albert Ludwigs University Freiburg, Freiburg, Germany; University Freiburg, Germany

## Abstract

The proinflammatory cytokine TNFα fails to provoke cell death in isolated hepatocytes but has been implicated in hepatocyte apoptosis during liver diseases associated with chronic inflammation. Recently, we showed that TNFα is able to sensitize primary murine hepatocytes cultured on collagen to Fas ligand-induced apoptosis and presented a mathematical model of the sensitizing effect. Here, we analyze how TNFα induces apoptosis in combination with the transcriptional inhibitor actinomycin D (ActD). Accumulation of reactive oxygen species (ROS) in response to TNFR activation turns out to be critical for sustained activation of JNK which then triggers mitochondrial pathway-dependent apoptosis. In addition, the amount of JNK is strongly upregulated in a ROS-dependent way. In contrast to TNFα plus cycloheximide no cFLIP degradation is observed suggesting a different apoptosis pathway in which the Itch-mediated cFLIP degradation and predominantly caspase-8 activation is not involved. Time-resolved data of the respective pro- and antiapoptotic factors are obtained and subjected to mathematical modeling. On the basis of these data we developed a mathematical model which reproduces the complex interplay regulating the phosphorylation status of JNK and generation of ROS. This model was fully integrated with our model of TNFα/Fas ligand sensitizing as well as with a published NF-κB-model. The resulting comprehensive model delivers insight in the dynamical interplay between the TNFα and FasL pathways, NF-κB and ROS and gives an example for successful model integration.

## Introduction

Hepatocyte apoptosis is associated with many acute and chronic liver diseases. Regulation of the apoptotic process is complex and mainly triggered through the activation of so-called death receptors [1]. Among these, the tumor necrosis factor (TNF)-receptor-1 and its ligand TNFα play a crucial role [2]. Upon binding of TNFα to TNFR1, a membrane bound complex I consisting of TNFR1, RIP-1, TRAF2 and TRADD is first formed which rapidly activates the survival transcription factor NF-κB and the c-Jun N-terminal kinase (JNK) [3]. NF-κB activation is the main reason why TNFα alone does not primarily lead to hepatocyte apoptosis. However, to signal for cell death, a second, receptor-free complex II has to assemble in the cytoplasm which still contains RIP1, TRAF2 and TRADD but recruits FADD and procaspase-8 [3]. TNFR1 activation alone only induces a weak and transient formation of complex II [2]. At complex II procaspase-8 is activated by autocatalytic cleavage [4]. Active caspase-8 can then trigger two different apoptotic signaling pathways. In the so-called type I cells, active caspase-8 directly cleaves and activates procaspase-3 to induce efficient cell death execution. In so-called type II cells, caspase-8 preferentially processes the BH3-only protein Bid into its truncated form tBid. tBid belongs to the subclass of BH3-only Bcl-2 family members such as Bim, Puma, Noxa, which sense apoptotic stimuli and convey the death signals to Bax/Bak activation on mitochondria [5]. Bax/Bak are essential for mitochondrial membrane permeabilisation (MOMP) and the release of apoptogenic factors such as cytochrome c. Cytochrome c finally activates the Apaf-1/caspase-9 apoptosome resulting in effector caspase-3/-7 activation and cell death execution. However, it has been shown that Bid-independent pathways can also lead to mitochondrial activation [6]. Recently, a study demonstrated that besides Bid the BH3-only protein Bim is also essential for TNFα-induced apoptosis in hepatocytes [7].

While the initiation of apoptosis by the receptor complex and the execution via the mitochondrial pathway constitute two parts of the machinery, several other pathways affect the apoptotic process dramatically. Among these, the JNK pathway deserves special interest. JNK is a serine/threonine protein MAPK which is known to be rapidly activated by TNFα complex I via the MAPK kinase MKK7 [8], [9]. This transient activation is normally terminated by MAPK phosphatases (MKPs) within 60 minutes, a mechanism itself controlled by NF-κB survival signaling [10]. However, under certain conditions TNFα can lead to a sustained activation of JNK which causes apoptosis [11]. To induce sustained JNK activation, the termination mechanism of the JNK activity is deranged, namely the MKPs are inhibited by reactive oxygen species (ROS). TNFα-induced ROS accumulate as soon as they are no longer suppressed by antioxidant enzymes, such as superoxide dismutases (SODs), glutathione peroxidase or ferritin, and can then cause oxidation and inhibition of MKPs [10], [12]. When, under the explained conditions, ROS induce a prolonged activation of JNK, several mechanisms linking this pathway to cell death execution have been proposed. It has been reported that JNK directly activates the E3 ubiquitin ligase Itch which induces proteasomal processing of cFLIP [13]. cFLIP specifically inhibits caspase-8 by interacting with FADD, therefore preventing the activation of procaspase-8 at complex II as well as at the DISC [14]. If cFLIP is degraded by JNK and Itch, complex II can recruit procaspase-8 and execute the apoptotic process as described above. Furthermore, it has been proposed that JNK can directly activate the mitochondrial pathway. While it has been suggested that JNK generates the proapoptotic Bid cleavage product jBid [15] several other reports indicate that JNK activates the BH3-only protein Bim [7], [16]. Both Bid and Bim can then activate the mitochondrial apoptosis pathway.

As mentioned above, TNFα alone does not lead to cell death and this is due to its strong NF-κB activating properties. NF-κB survival signaling interferes with the TNF pathway at various levels to prevent apoptosis. Hereby, the antiapoptotic activity of NF-κB mainly depends on gene induction, namely the transcriptional regulation of survival genes [17]. Among these, the caspase inhibitors cFLIP and XIAP as well as the inhibitor of apoptosis proteins, cIAP1 and 2 and the Bcl-2 family proteins are well known to be upregulated by NF-κB in several cell types [4], [18]. However, these antiapoptotic proteins alone cannot fully protect the cells from apoptosis. Thus, another NF-κB-dependent mechanism has been suggested such as the prevention of ROS accumulation [12]. In contrast, it was shown that the proapoptotic effects of JNK do not require transcription and that JNK1 activates a mitochondrial death pathway [19]. Taken together, apoptosis induction by TNFα is regulated by a complex network of signaling pathways and understanding these processes requires a holistic approach which integrates all levels of the network. Accordingly, mathematical modeling also needs to integrate the different levels of the network to describe the biological process properly and thereby allow reliable predictions.

In this study, we introduce a mathematical model for TNFα-induced apoptosis in hepatocytes which is based on presented measurement results and literature data. The developed model is connected with a TNFα/Fas ligand sensitizing model [20] and with an NF-κB model [21] to reflect the complex crosstalks in this system. The final comprehensive model covers the sensitizing effect of TNFα towards FasL-induced apoptosis as well as apoptosis after TNFα plus ActD or plus cycloheximide, respectively, and gives an important example for successful model integration. All models employed in this study are based on ordinary differential equations (ODEs) and mass action kinetics. [20], [22]

## Materials and Methods

### Mice

Wild-type mice were purchased from Jackson Laboratories. Bid−/− mice and XIAP−/− were generously provided by Andreas Strasser and John Silke, WEHI, Melbourne, respectively. All mice were bred on the C57BL/6 background for several generations. Hepatocyte isolation from these mice was approved by the animal experimental committees and animals were handled and housed according to specific pathogen free (SPF) conditions.

### Isolation and cultivation of primary mouse hepatocytes

Primary hepatocytes were isolated from 8–12 week old B6 (C57Bl/6NNrl) mice using the collagenase perfusion technique as previously described [23].

### Preparation of cytosolic and mitochondrial lysates

Crude mitochondrial and cytosolic fractions were prepared as described before [22]. Briefly, 6×10^6^ detached hepatocytes were resuspended in SEM buffer (10 mM Hepes-KOH and 250 mM sucrose) containing protease inhibitors as described above and homogenized in a Dounce homogenizer. The nuclei were removed by centrifugation at 500×g, and crude mitochondria were obtained from the supernatant by an additional centrifugation step at 10'000×g. Mitochondria were washed twice in SEM buffer and then solubilized in H8 buffer (20 mM Tris-HCl, pH 7.5, 2 mM EDTA, 2 mM EGTA, 6 mM ß-mercaptoethanol) containing 1% SDS. All fractions were used for Western Blot analyses; cytosolic fractions were also used for cytochrome c detection by ELISA.

### DEVDase assay

The activity of the executioner caspases-3/-7 was measured by the fluorogenic DEVDase assay. Briefly, 1×10^6^ primary mouse hepatocytes were incubated with TNFα (R&D Systems) or actinomycin D (ActD) (Alexis) at different doses for the indicated times. Then the cell suspension was centrifuged, washed with PBS and homogenized in 50 µl homogenization buffer (30 mM Hepes-KOH, 2.5 mM MgCl_2_, 2.5 mM EGTA, 12 mM DTT supplemented with the protease inhibitors 12 µg/ml aprotinin, 12 µg/ml leupeptin, 0.5 µg/ml pepstatin, 0.125 µM PMSF, 1.5 µg/ml cytochalasin B). The caspase-3/-7 activity assay was performed exactly as described in [24] using the caspase-3 substrate DEVD-AMC (Alexis) at a concentration of 200 nM. Relative fluorescence units (RFU) values were calculated via the ratio of average rate of the fluorescence increase and protein concentration determined by Bradford assay (Biorad). To compare different experiments, RFU sample values were referred to negative control (untreated cells).

### Western blotting

50–70 µg of total protein was separated by SDS-PAGE (12%, 15% or 17,5% gels) and transferred to a 0.45 µm or 0.2 µm pore size PVDF membrane (Roche Applied Science and BioRad, respectively). The respective antigens were detected by antibodies against phospho-JNK at 1∶1000 (Cell Signaling), JNK at 1∶1000 (Cell Signaling), β-actin at 1∶10000 (MP Biomedicals) cFLIP_L/S_ and cFLIP_L_ both at 1∶1000 (Santa Cruz Biotechnology) and MnSOD at 1∶5000 (R&D Systems) followed by the respective horseradish peroxidase-labeled secondary antibodies (Jackson ImmunoResearch Laboratories or Cell Signaling), and the ECL plus chemiluminescence detection reagent (Amersham Biosciences). Chemiluminescent images were quantified using the LumiImager and the LumiAnalyst Software (Roche Applied Science).

### RNA isolation, cDNA synthesis and qRT-PCR

Total RNA was isolated using the RNeasy Plus Kit of Qiagen, according to the manufacturer's directions. The quantity and purity of RNA was determined by measuring the optical density at 260 and 280 nm. Subsequently, 1 µg of total RNA was converted to single strand cDNA using Quantiscript Reverse Transcriptase (Qiagen). The analysis of mRNA expression profiles was performed with multiplex quantitative real time PCR. In a 25 µl PCR reaction, 2 µl of cDNA (corresponding to 20 ng of total RNA input) was amplified in an Light Cycler 480 (Roche) using 2-fold QuantiTect Multiplex PCR Master Mix (Qiagen), 50 nM primers and 100 nM probe for the 18S rRNA reference gene (fwd: 5′-CGGCTACCACATCCAAGG-3′, rev: 5′-CGGGTCGGGAGTGGGT, probe: 5′-TTGCGCGCCTGCTGCCT), and 300 nM primers and 100 nM probe for the gene of interest. The following target gene primers and probes were used (all from Sigma Genosys): mouse cFLIP (fwd: 5′-TGCCAGAGTGTGGAGAACAG-3′; rev: 5′-TTACCCAGTCGCATGACAAA-3′; probe: 5′-GGGGGAGGTTATCTACCAAGT-3′) and mouse MnSOD (fwd: 5′- CCCTTAGGGCTCAGGTTTGTC-3′, rev: 5′-GCCACCGAGGAGAAGTACC-3′, probe: 5′- AGATGTTACAACTCAGGTCGCTCTTCAG-3′). The mRNA level for the gene of interest was determined as 2-ΔΔCT and reflects changes relative to untreated cells.

### Cytochrome c ELISA

To quantify the amount of cytochrome c in subcellular fractions an ELISA kit from R&D Systems was applied. From 6×10^6^ hepatocytes cytosolic fractions were obtained as described above and ELISA performed according to the manufacturer's instructions. Briefly, 1∶50 dilutions from cytosolic fractions and corresponding standards were analyzed and absorbance measured at 450 nm. Cytochrome c concentrations were calculated using the standard concentration curve.

### Measurement of reactive oxygen species by dichlorofluorescin fluorescence assay

ROS produced by the different treatments with TNFα (R&D Systems), ActD (Alexis) or butylhydroxyanisol (BHA) (Sigma) were assessed by the oxidation-sensitive probe 2, 7-dichloro-dihydrofluorescin diacetate (H_2_DCFDA, Sigma). H_2_DCFDA diffuses easily across the cell membrane and is then trapped in the cell by deacetylation. In the presence of ROS, H_2_DCF is rapidly oxidized to the highly fluorescent compound dichlorofluorescin (DCF) [25]. Briefly, 1×10^6^ primary mouse hepatocytes were incubated with TNFα, ActD or BHA for the indicated times and H_2_DCFDA 20 µM was added in the last 20 minutes of incubation. After centrifugation at 2150×g, 4°C for 3 min, washing with PBS, another centrifugation cells were lysed in 70 µl lysis buffer (20 mM Tris/HCl pH 7.4, 136 mM NaCl, 2 mM EDTA, 10% glycerol, 4 mM benzamidine, 50 mM β-glycerophosphate, 20 mM Na-diphosphate, 10 mM NaF, 1 mM Na_3_VO_4_, 1% Triton X-100 supplemented with the protease inhibitors 5 µg/ml aprotinin, 5 µg/ml leupeptin, 0.2 mM AEBSF) by shaking at 4°C for 20 min followed by a final centrifugation at 20'800×g, 4°C for 10 min. 10 µl of cell lysate was diluted with 90 µl of assay buffer (100 mM HEPES-KOH supplemented with 100 mM DTT) in a 96-well plate and fluorescence was determined using an excitation wavelength of 485 nm and emission of 540 nm. To quantify fluorescence a DCF standard curve was measured and calculated as described before [26] and sample values determined as fold increase referred to untreated control.

### Other experimental procedures

Other experimental procedures are described in detail in the Supplementary Information S1. These include detailed description of the isolation and cultivation of primary murine hepatocytes, cultivation of 3T9 mouse embryonic fibroblasts (MEFs), preparation of total and nuclear extracts and electrophoretic mobility shift assay (EMSA).

## Results

### TNFα induces apoptosis via type II extrinsic pathway but only in the presence of a transcription inhibitor

**Figure 1:**
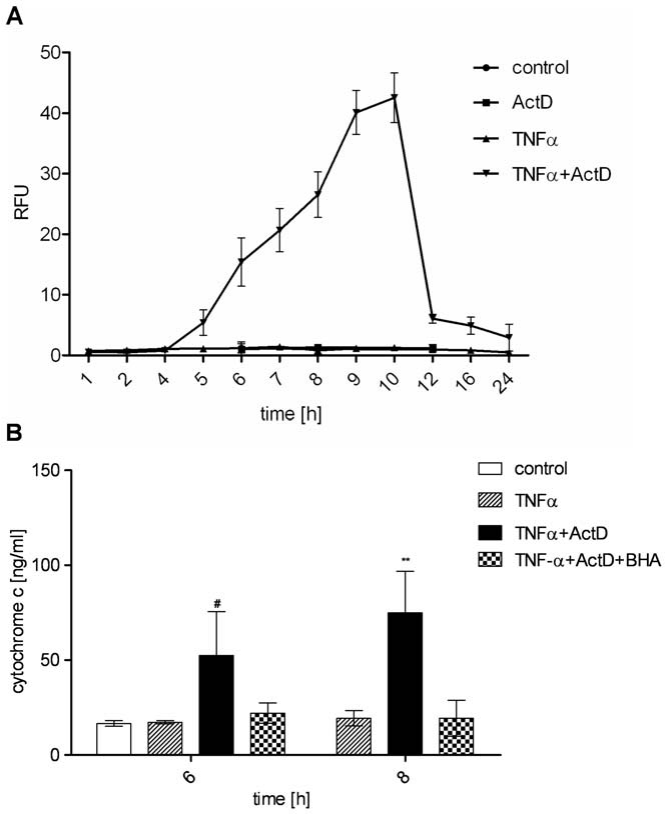
TNFα and ActD induce strong caspase-3/-7 activation and antioxidant-sensitive cytochrome c release in primary hepatocytes. (A) Caspase-3/-7 activity determined by fluorogenic DEVDase assay of primary murine hepatocytes treated with TNFα (25 ng/ml), ActD (0.4 µg/ml) or TNFα+ActD. (B) Cytochrome c concentration determined by ELISA in the cytosol of hepatocytes after treatment with TNFα or TNFα+ActD in the presence or absence of the antioxidant BHA (100 µM) for the indicated times. Means of at least three independent experiments ± s.d. are shown (** P<0.01, # P<0.05 both versus TNFα-treated cells, Student's t-test).

In order to characterize the TNF apoptosis pathway in hepatocytes, we first examined the dynamics of effector caspase-3 activation. While TNFα alone does not activate caspase-3, hepatocytes pretreated with the transcription inhibitor ActD show a remarkable increase in caspase-3/-7 activity within 5–10 h of treatment (Figure 1A), consistent with previously reported results [19]. Caspase-3/-7 activation resulted in cell death as we have previously reported [22] and therefore reflects a measure for apoptosis quantification. Furthermore, we have demonstrated before in hepatocytes from Bid^−/−^ mice that TNFα-induced apoptosis is clearly dependent on the BH3-only protein Bid indicating that apoptosis occurs via type II pathway. We substantiated this observation by demonstrating that loss of the caspase-3 and -9 inhibitor XIAP does not further enhance cell death after TNFα and ActD treatment [22]. In the type II apoptosis pathway XIAP is neutralized by Smac/DIABLO which is released from the mitochondria. Therefore, loss of XIAP does not lead to any further increase of caspase-3 activation as it is observed in type I pathway. Accordingly, XIAP^−/−^ hepatocytes did not reveal an increased caspase-3 activity after TNF-α and ActD treatment compared to wt hepatocytes [22]. To confirm that TNFα induces apoptosis via type II pathway we investigated another downstream event of mitochondria activation which is cytochrome c release to the cytosol. Indeed, we found cytochrome c to be released from the mitochondria in a time-dependent manner as shown in Figure 1B. The above described results clearly show that TNFα induces type II apoptosis pathway via the proteins Bid and cytochrome c in the presence of the transcription inhibitor ActD.

### ROS accumulate upon TNFα treatment and mediate mitochondria-dependent cell death

**Figure 2:**
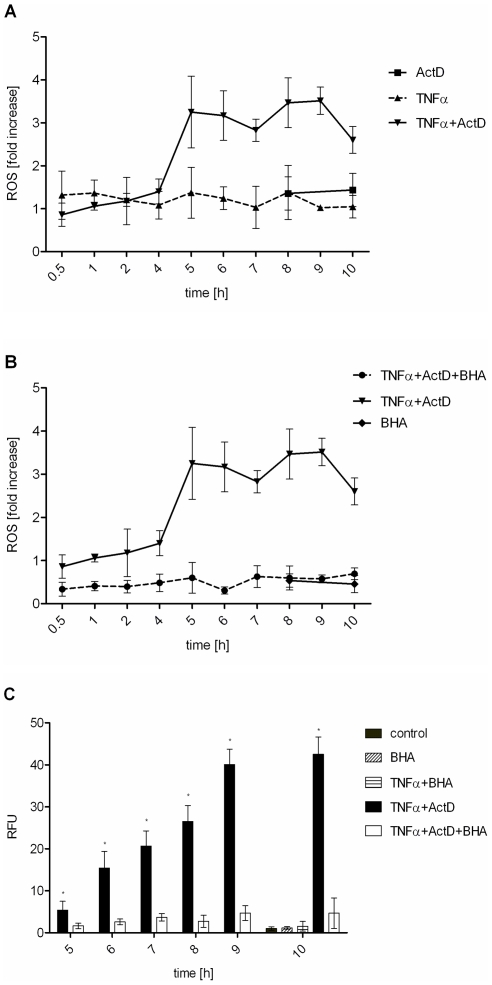
TNFα plus ActD induce antioxidant-sensitive ROS accumulation which is critical for caspase-3/-7 activation. (A) Hepatocytes were treated with TNFα (25 ng/ml), ActD (0.4 µg/ml) or TNFα+ActD and ROS measured by dichlorofluorescin fluorescence assay and referred to untreated control. (B) ROS accumulation determined when cells were preincubated with the antioxidant BHA (100 µM) for 30 minutes before TNFα+ActD treatment. (C) Caspase-3/-7 activation of cells treated with TNFα, TNFα+ActD or both preincubated with BHA. Values represent means of at least three independent experiments ± s.d. (* P<0.001 versus TNFα+ActD+BHA-treated cells, Student's t-test).

Since oxidative burst has been implicated in apoptosis under several circumstances, we investigated ROS accumulation upon TNFα treatment. Again, TNFα alone did not induce any significant increase in intracellular ROS amounts. Interestingly, the cells showed elevated ROS levels after 5 hours of TNFα and ActD treatment remaining on that level over the whole incubation time investigated (Figure 2A). Consequently, preincubation of primary murine hepatocytes with the antioxidant butylhydroxyanisol (BHA) before TNFα and ActD challenge reduced ROS to the level of untreated control cells (Figure 2B). Note, that basal ROS were also reduced by BHA. Furthermore, we investigated if the observed ROS accumulation plays a critical role in TNFα mediated apoptosis. Therefore, primary murine hepatocytes were again preincubated with BHA and apoptosis was monitored by measuring capase-3/-7 activation. Indeed, the presence of an antioxidant which neutralizes ROS (Figure 2B) rendered the cells completely from apoptosis as shown in Figure 2C. Interestingly, BHA also prevented TNFα and ActD-induced cytochrome c release from the mitochondria (Figure 1B). This indicates that ROS are responsible for cytochrome c release during mitochondria-dependent apoptosis.

### Sustained JNK activation due to ROS accumulation is critical for apoptosis induction

**Figure 3:**
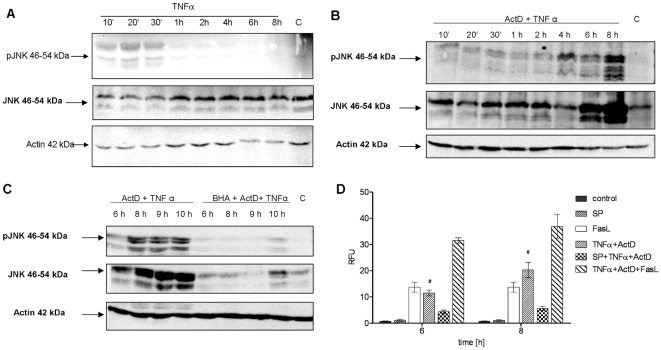
Sustained activation of JNK after TNFα plus ActD is ROS-dependent and involved in caspase-3/-7 activation. (A) Western blot analysis of JNK and phospho-JNK (P-JNK) after treatment with TNFα (25 ng/ml) for the indicated times. (B) JNK and P-JNK levels determined by Western blot analysis in cells treated with TNFα (25 ng/ml) and ActD (ActD) (0.4 µg/ml) for the indicated times. (C) Analysis of JNK and P-JNK by Western blot after treatment of hepatocytes with TNFα+ActD with or without preincubation with BHA 100 µM for the indicated times. Actin is used as loading control. One representative experiment is shown. (D) Caspase-3/-7 activation of cells treated with TNFα+ActD with or without preincubation with the JNK inhibitor SP600125 20 µM. For comparison, cells were also treated with FasL (50 ng/ml) for 6 hours with or without costimulation with TNFα+ActD for the indicated times. Means of at least three independent experiments ± s.d. are shown (# P<0.05 versus TNFα+ActD-treated cells, Student's t-test).

ROS are known to modulate the activity of the TNF receptor activated kinase JNK. Since we observed that ROS mediate cell death in murine hepatocytes, we next examined the activity of JNK by Western Blotting of the active form phospho-JNK. As expected, JNK was slightly activated by TNFα alone and in combination with ActD in the first 30 minutes (Figure 3A and 3B) concomitant with previously reported results [20]. Notably, TNFα and ActD strongly induced sustained JNK activation at 6–8 h of incubation (Figure 3B) which is exactly the time when maximal caspase activation can be observed. Underlining the relevance of this sustained JNK activation for apoptosis, it turned out to be sensitive to antioxidants. The antioxidant BHA effectively prevented the later formation of pJNK as shown in Figure 3C. Astonishingly, TNFα and ActD treatment strongly upregulated the amount of JNK protein (Figure 3B) which did not occur with TNFα alone (Figure 3A) and was prevented by the antioxidant BHA (Figure 3C). This suggests that TNFα and ActD induce sustained phosphorylation of JNK at least in part by upregulating the inactive, unphosphorylated form JNK and that ROS mediate this upregulation. To finally substantiate the role of JNK in TNFα-mediated apoptosis, we investigated the influence of JNK inhibition on caspase-3/-7 activation. Preincubation of hepatocytes with the JNK inhibitor SP600125 strongly reduced caspase-3/-7 activity and protected the cells from cell death (Figure 3D) which is in accordance with [19]. In addition, TNFα plus ActD-mediated caspase activation could be further elevated by additional incubation with the death receptor ligand Fas. Together with previous observations, these findings indicate the direct involvement of ROS-mediated sustained JNK activation in apoptosis induction by TNFα plus ActD.

### TNFα activates NF-κB and upregulates the antiapoptotic genes cFLIP and MnSOD but fails to increase the respective protein levels

**Figure 4:**
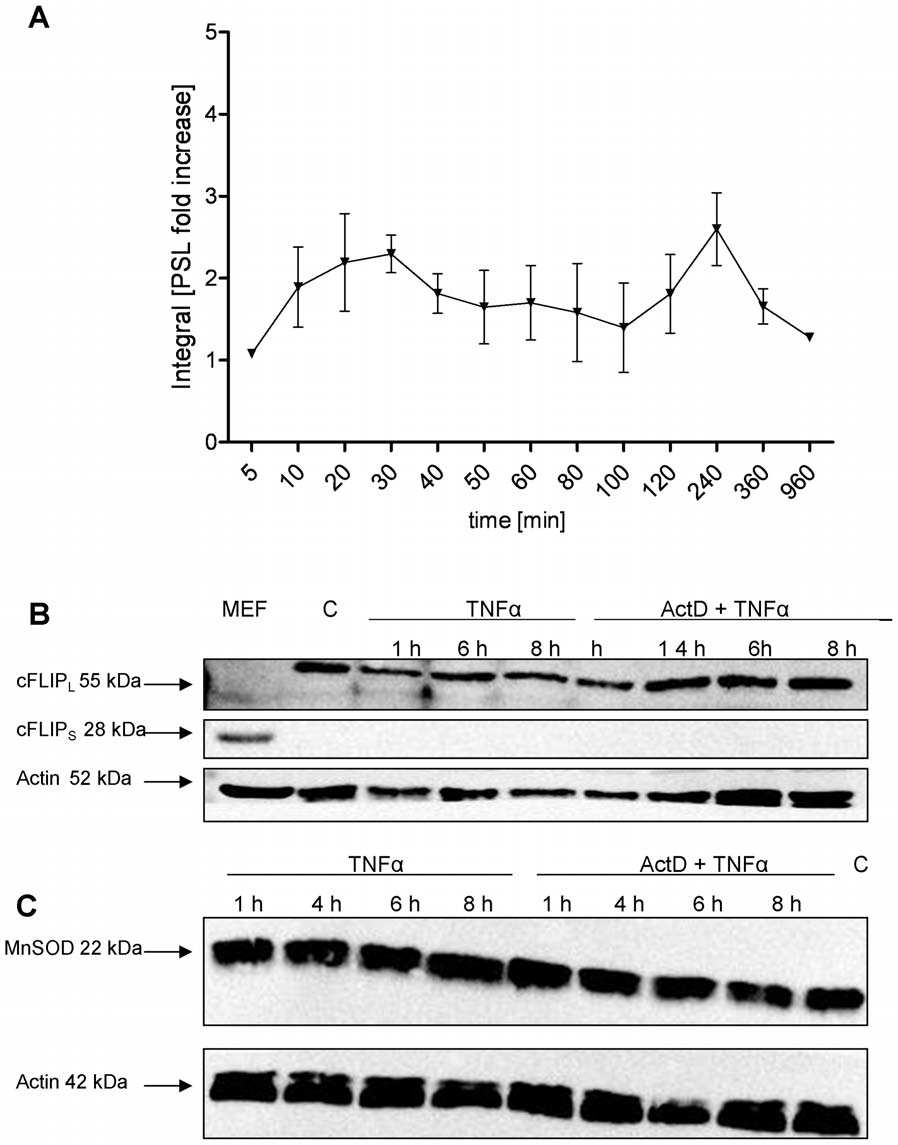
TNFα induces NF-κB activation but fails to upregulate cFLIP and MnSOD protein. (A) Kinetics of NF-κB-DNA binding activity measured by EMSA in hepatocytes treated with TNFα (25 ng/ml) for the indicated times. Means of at least three independent experiments ± s.d. are shown. cFLIP_L/S_ (B) and MnSOD (C) protein levels determined by Western Blot analysis in primary murine hepatocytes and mouse embryonic fibroblasts (B, left panel) treated with TNFα (25 ng/ml) with or without ActD (0.4 µg/ml) for the indicated times. Actin is used as loading control. One representative experiment is shown.

We have shown that TNFα only induced apoptosis in the presence of the transcription inhibitor ActD. One of the main antiapoptotic transcription factors which is known to prevent TNFα-mediated apoptosis is NF-κB. Therefore, we first tested by EMSA whether NF-κB is activated by TNFα and which dynamics can be observed. As expected, TNFα leads to an early NF-κB induction phase in the first 30 minutes followed by a second induction between 4 and 6 h (Figure 4A). Both NF-κB activation phases lead to transcriptional upregulation of a variety of target genes. Among these, several antiapoptotic genes have been implicated in preventing apoptosis in response to TNFα. Consequently, we examined the expression of the antiapoptotic genes cFLIP and MnSOD by qRT-PCR. Whereas MnSOD mRNA showed a very low induction at 6–8 h, cFLIP revealed a moderate upregulation 6 h after TNFα treatment. mRNA upregulation of both genes was completely blocked by ActD (Figure S1A and S1B). However, Western Blot analysis revealed that neither MnSOD nor cFLIP protein levels were increased by TNFα treatment or decreased in response to TNFα and ActD treatment at the times when caspase-3/-7 activation was observed (Figures 4B and 4C). This result suggests that besides MnSOD, other NF-κB target genes might play a more prominent role in preventing ROS accumulation as it has been suggested before [27], [28]. Interestingly, this finding shows that cFLIP degradation is not involved in TNFα and ActD-induced apoptosis. However, several reports indicate that it is crucial in TNFα and cycloheximide-induced hepatocyte apoptosis [13]. We could confirm the TNFα and cycloheximide-mediated cFLIP degradation in our experimental setting (data not shown). These observations strengthen the role of NF-κB in preventing TNFα-induced apoptosis and support the idea that, according to the respective costimulus, different proteins have to be considered trying to understand this complex apoptosis pathway.

### Modeling

**Figure 5:**
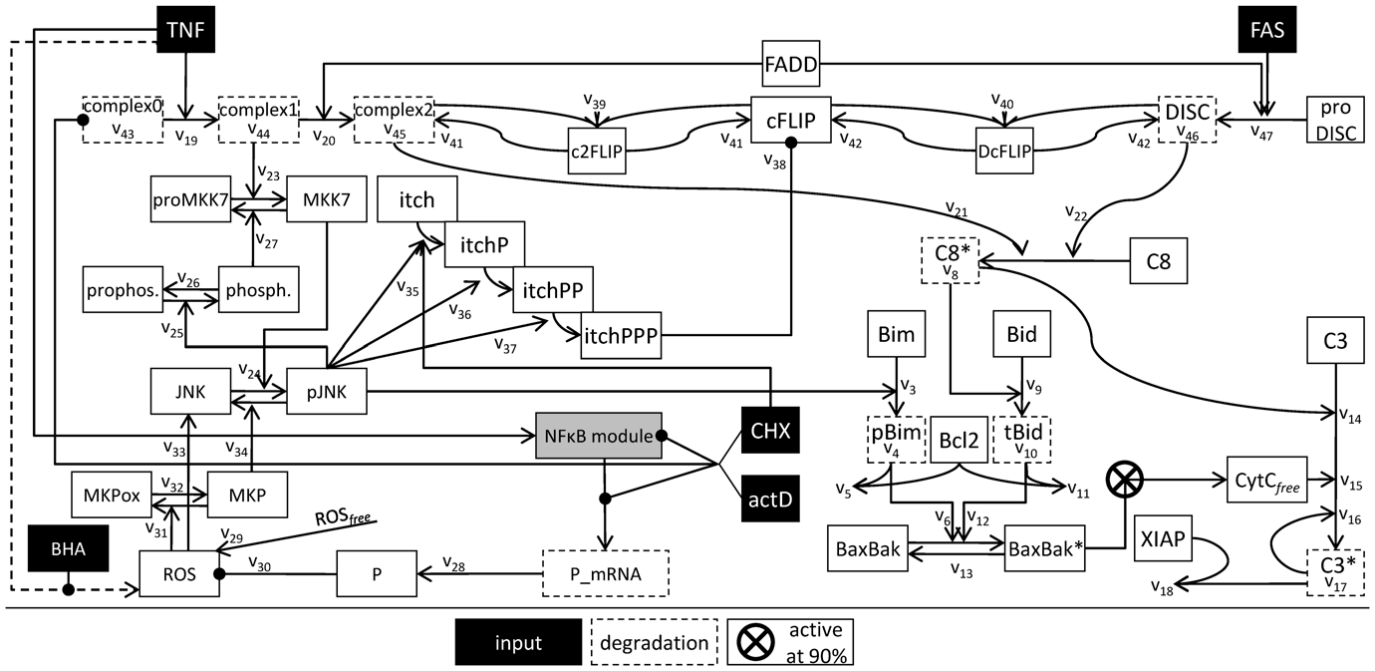
Schema of the mathematical TNFα-induced apoptosis model. Illustration of the model structure in accordance to the model equations introduced in the *Modeling* section. Input stimuli of the model are TNFα, Fas ligand, BHA, ActD (actD) and cycloheximide (CHX). The NF-κB model is depicted as grey box for clarity. The kinetic parameters are indicated to assign the equations to the schema. Production rates are written into the boxes for the according species. Species which are degraded in the model are framed with dashed lines.

**Figure 6:**
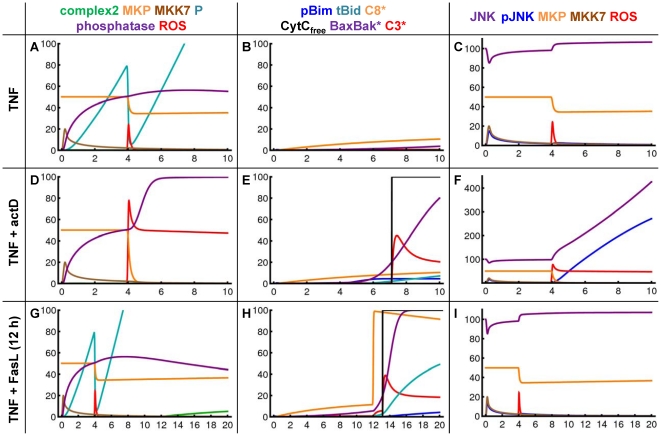
Simulation results of the TNFα-induced apoptosis model after TNFα, TNFα plus ActD and TNFα/Fas ligand sensitizing. Simulation results for pivotal species of the TNFα-induced apoptosis model after stimulation with TNFα only (A–C), with TNFα plus ActD (D–F) over 10 hours or after TNFα/Fas ligand sensitizing (12 h TNFα preincubation before Fas stimulus) (G–I) over 20 hours. The species are shown in separate panels for clarity as indicated in the legend.

**Figure 7:**
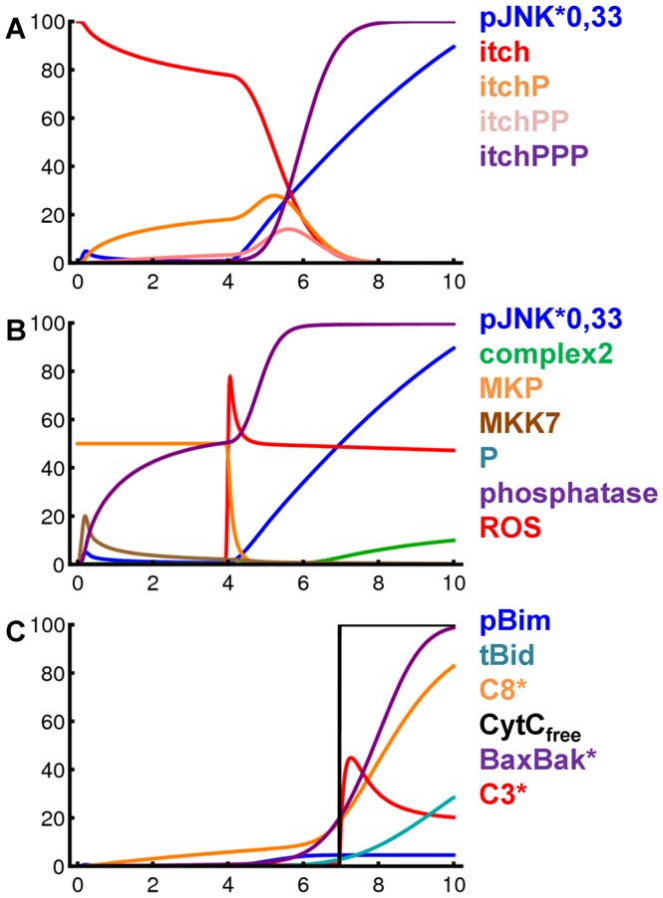
Simulation results of the TNFα-induced apoptosis model after TNFα plus cycloheximide. (A–C) Simulation results for pivotal species of the TNFα-induced apoptosis model over 10 hours after stimulation with TNFα plus cycloheximide. The amount of pJNK is reduced to one third for this Figure. The species are shown in separate panels for clarity as indicated in the legend.

Despite the detailed knowledge on individual molecule interactions underlying TNFα-induced apoptosis, it remains unclear if these interactions can explain the experimentally observed overall behavior of the system. Mathematical modeling is a powerful tool to address the challenges provided by the complexity of the pathways analyzed in the experiments. The overall model we present here starts from the recently published model describing the TNFα and Fas ligand sensitizing effect [20]. First the sensitizing model was enhanced to achieve connection sites for further interactions. Then an NF-κB model [21] was appended to deliver mRNA expression. Finally, the loop was closed backwards to the control of pJNK activity after TNFα stimulation. Figure 5 shows a scheme of the overall model. The model is available in the supplementary information (Model S2). Model S1 shows a diagram of the sensitizing model as presented in [20]. A scheme of the integrated NF-κB module can be found in [21]. Simulation results of the overall model are shown in Figure 6 and 7.

### The TNFα and Fas ligand sensitizing model is expanded to attach JNK and ROS signaling

The already existing sensitizing model is based on biological experiments including measurement of caspase-3/-7 activity and cytochrome c release under varying conditions, such as only TNFα, only Fas ligand and combination of both, and reproduces these data [20]. Since this model is used as initial point for the study presented here we will describe it shortly. The sensitizing model has two inputs, TNFα and Fas ligand. Active caspase-3 is considered as output and indication for apoptosis. Fas ligand activates caspase-8 and thereby also a limited amount of caspase-3 via the direct type I pathway. Caspase-3 is absorbed to a certain amount by its inhibitor XIAP. In addition, active caspase-8 cleaves Bid to its active form tBid and thereby activates a certain amount of Bax/Bak but not enough for mitochondrial membrane permeabilisation. Therefore, Fas ligand does not induce cytochrome c release from the mitochondria in the sensitizing model which was shown for cultured primary hepatocytes before [22]. TNFα directly leads to activation of JNK which phosphorylates Bim, a BH3-only protein in turn also able to activate Bax/Bak. However, TNFα alone does not activate sufficient amounts of these proteins to induce cytochrome c release in the sensitizing model and therefore does not result in any caspase-3 activation. In addition, Bcl2 is included in the model as an inhibitor for pBim and tBid. However, if TNFα and Fas ligand pathways crosstalk at an appropriate time their effects on Bax/Bak turn out to be additive, cytochrome c is released and a significantly higher number of caspase-3 is activated. This causes strong apoptosis which is called the sensitizing effect. As the mechanisms leading to mitochondrial pore formation and abrupt release of cytochrome c are not yet known in detail, the release is achieved in the model using a step function which is triggered at 90% activation of the Bax/Bak pool. Apart from this step function, the complete model is based on ODEs and mass action kinetics. The parameters identified for this model have arbitrary units. They deliver a proof of concept for the model as it fits the measurement data as well as current knowledge. Also the initial amounts of the involved molecular species are unknown and have been set to artificial numbers. Interestingly, the structure of the sensitizing model allows also simulating different cell types. TNFα can be enabled to induce apoptosis by changing initial amounts of proteins which is equivalent to a different expression pattern.

In the sensitizing model, TNFα directly drives the phosphorylation of JNK. This was sufficient to reproduce the sensitizing effect which is mainly based on BH3-only protein family members but is of course a strong simplification. In a first step, we expanded this area of the model to create connecting points for interaction with other components of the signaling pathway and allow for more complex signaling behavior. The TNFα input is now converting the species complex0 to complex1 (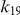) [4]. These species and also the species complex2 and DISC are modeled in a simplified manner as one species although they correspond to multi-protein complexes. The species complex0 is also constitutively produced and degraded to maintain its initial amount in an unstimulated setting (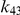). The Boolean variable 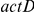 is explained below. The species complex1 forms complex2 by recruitment of FADD (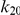) [4] and is also degraded over time (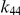). Thereby complex1 is realized as the main species before complex2 is formed after TNFα stimulus by 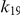 = 0.05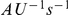 and 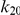 = 0.001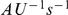. This is in accordance with JNK signalling being the main function of TNFα in the model whereas only weak caspase-8 activation at complex2 occurs after TNFα.
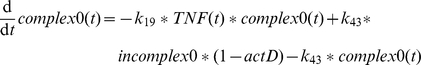


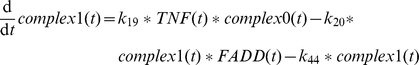
In addition, complex1 activates proMKK7 to MMK7 (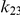) [9]. Active MKK7 phosphorylates JNK to pJNK (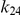) [9]. The pJNK protein in turn activates a phosphatase (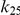) to finally inactivate MKK7 again (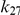) as it has been shown for the p38 regulation cascade [29]. It is not yet known which phosphatase performs this task. We assume that the phosphatase recovers to prophosphatase over time (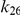). The dephosphorylation of pJNK is accomplished by MKPs (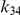) [10], [30], whose regulation is discussed later on. As these proteins establish a self terminating mutual activation chain the model reproduces the disappearance of pJNK after 30 minutes (Figure 3A and 3B). In addition, the experimentally observed relation between ROS and the amount of JNK protein is modeled by a linear relation (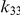) as best approximation to our knowledge. Analysis of Figure 3B reveals an increase of overall JNK after 8 hours by factor 5 after TNFα plus ActD stimulus. Accordingly, the parameter 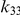 is set to 2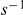 which results in an increase from 100 to 507 overall JNK species in the model after 8 hours in this scenario.
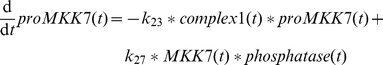


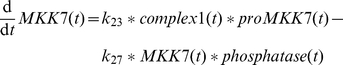


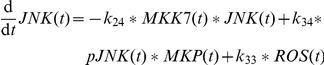


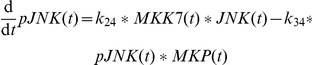


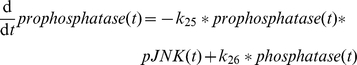


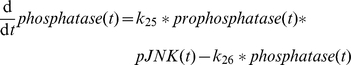
Due to these changes in the model the parameters 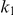 and 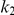 from the sensitizing model dropped out. These are the only structural changes of the original model we made during the integration process. Moreover, the initial values for all species remain unchanged. Only the parameters 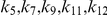 are newly fitted as shown in the supplementary information (Model S1) to maintain proper reproduction of the sensitizing effect inside the new, extended model framework. Additionally, the threshold of Bax/Bak activation for mitochondrial pore formation was lowered from 90% to 20% as this process is now more tightly regulated in the extended model. This is in accordance with a recent study suggesting an only weak increase of Bax in the mitochondria before pore formation [31].

In the sensitizing model, TNFα and Fas ligand signals do not crosstalk upstream from Bax/Bak. This is now adjusted, since caspase-8 is not only activated by Fas ligand via the DISC (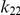) but also at complex2 after TNFα (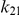) [4]. Active caspase-8 is also degraded over time (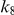). Due to these changes in the model the parameter 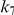 from the sensitizing model dropped out. The influence of Fas ligand is now modeled as activating the formation of the DISC from species subsumed as proDISC and recruiting FADD (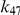). Thereby FADD is a common pool for complex2 and DISC formation.
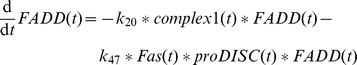


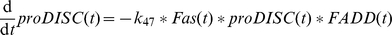


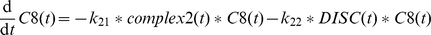


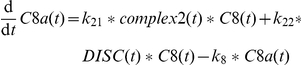
The activation of caspase-8 at complex2 as well as at the DISC is competitively inhibited by cFLIP whose main type in murine hepatocytes is the long form according to our experimental results. The species cFLIP binds complex2 in a complex named c2FLIP in the model (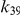) and binds DISC in a complex named DcFLIP in the model (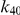) [14]. These complexes are also dissociating to their components again (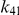 and 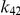). Finally, complex2 and DISC are degraded (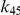 and 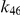).
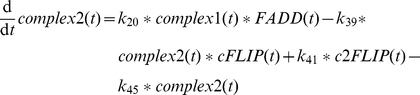


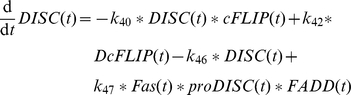


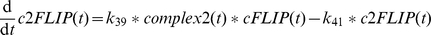


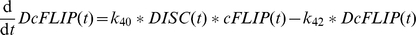
The amount of free cFLIP species in the model can be regulated by several influences. It is affected by c2FLIP and DcFLIP formation. According to our results in primary murine hepatocytes, the amount of cFLIP protein is stable after TNFα plus ActD treatment. This result indicates that the cFLIP protein is not degraded or produced from mRNA in the considered time frame under the according experimental conditions. However, cFLIP was reported to be degraded by the enzyme Itch (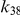) when cells were treated with TNFα plus cycloheximide [13].
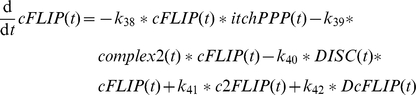
The activation of Itch has been reported to be driven by pJNK, where several phosphorylations are required (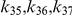). We assume three times phosphorylated Itch (itchPPP) as active species as supposed in [32]. Itch can function as an integrator for pJNK. As illustrated in Figure 7A the catenation of several steps allows for a decoupling of the curve shapes of pJNK and itchPPP. To achieve this effect the kinetic parameters for the successive phosphorylation steps need to be increasing which is consistent with a biological cooperativity effect. The values have been chosen as 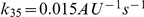, 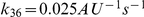 and 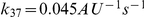. As Itch was shown to be a highly stable protein [33] the model does not include Itch degradation. Overall, Itch couples JNK activation to cFLIP in the model which was shown as principle mechanism after TNFα plus cycloheximide treatment [13]. As this Itch-mediated cFLIP degradation does not occur after TNFα plus ActD treatment the model discriminates between ActD and cycloheximide. Itch can only be phosphorylated and activated in the presence of cycloheximide represented by a Boolean variable named 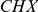. Without cycloheximide the proapoptotic impact of pJNK in the model is exerted solely via phosphorylation of Bim as modelled in [20].
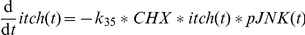


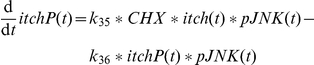


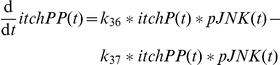


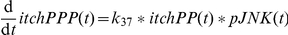



### An NF-κB model is attached to the TNFα input signal

An NF-κB model is included as module in the TNFα signaling pathway model to allow description of transcriptional effects on the network. We used the NF-κB model from Lipniacki *et al.* [21] because of its high grade of accuracy. The model provides a detailed description of NF-κB activation in response to TNFα and delivers the resulting mRNA production. It includes the proteins NF-κB, IKK, IκBα, A20 and several complexes of them as well as transcripts of A20, IκBα and an additional exemplary target gene of NF-κB. Cytoplasm and nucleus are modeled as separate compartments. Regarding the initial amounts of proteins and mRNA species Lipniacki *et al.* used several previous studies which measured components of the NF-κB signaling pathway in absolute units. The model equations contain 30 parameters from which 5 are taken from the literature, 13 are assumed, 10 were fitted and the value of 2 parameters turned out to be insignificant. Using these parameters the model reproduces oscillations of unbound nuclear NF-κB proteins and the related species which is considered to be typical for this biological system. The model is adopted here with very few changes that are described in the following section.

Notation for parameters and species is retained unchanged from Lipniacki *et al.* for better comparability. The input of the NF-κB model is realized by Lipniacki *et al.* with a logical variable named 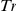 [21]. This is maintained by setting 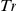 to “1” if the cell is stimulated with TNFα and to “0” if not. We newly introduce a logical variable for gene expression to realize the impact of transcription inhibitor ActD in this module. This variable is named 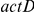 and is set to “1” in presence and set to “0” in absence of ActD. The modeling of ActD without interstages is based on our experimental setting because ActD is added 30 min prior to TNFα stimulation. The latter is equivalent to time point zero in the model because transcription is already completely stopped at the beginning of stimulation. By introducing ActD the following equations of the model from Lipniacki *et al.* [21] are changed so that there is not any transcript production in the presence of ActD.
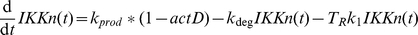


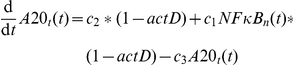


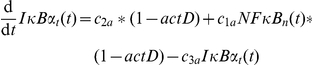
All other equations and parameter values of the NF-κB model remained with a single exception. We assume the relation of cytoplasmic to nuclear volume in hepatocytes to have value 3 instead of 5 as used for fibroblasts by Lipniacki *et al.* [21]. In addition, as the original time unit in the Lipniacki model are seconds, all parameter values are multiplied with 3600 to achieve hours as consistent unit in the overall model. As initial values of the state variables their steady state values from unstimulated model simulation is calculated.

The output of the NF-κB model from Lipniacki *et al.* is an unspecified exemplary NF-κB target gene [21]. We use the according equation to model the mRNA for a protein P.
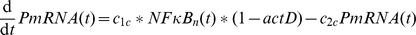
The parameter values regarding regulation of the transcripts are changed in comparison with the target gene from Lipniacki *et al.* to fit our measurement data. We applied an iterative manual fitting approach as also described by Lipniacki *et al.* [21]. The parameter 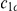 describes inducible mRNA synthesis and is fitted to the measurement data as 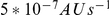. As only the amount of ROS released in addition after TNFα and not constitutively produced ROS is considered, accordingly only the amount of its counterpart P additionally induced via NF-κB is respected. By setting the variable 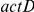 to zero the model can reproduce the fact that mRNA synthesis is completely blocked in the presence of ActD (Figure 5 and S1). The parameter 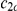 describes mRNA degradation and is set to 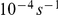. The translation rate 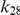 from mRNA to protein is set to 25 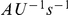.

Note that there is no NF-κB activation after Fas ligand in the model. This is in accordance to previous experimental findings in primary mouse hepatocytes which showed that there is only weak NF-κB activation and no induction of the NF-κB target genes such as cFLIP and cIAP2 in response to FasL [34].

### The regulation of ROS rules the network by controlling the amount of phosphorylated JNK

ROS can be judged as central players of the network. We will first discuss their origin and regulation and afterwards their effects. The origin of ROS due to TNFα stimulation are the mitochondria [35], [36]. We assume that the constitutively produced amount of ROS in the cell is not responsible for their effects after TNFα stimulation. Therefore, we decided to model only the additional amounts of ROS in this setting. Accordingly the initial value of ROS in the model is 0. This also suits our experimental approach measuring the total amounts of ROS in hepatocytes referred to untreated control. The data show that ROS accumulate after 4 hours and remain constant after 5 hours (Figure 2A). Recently, a mathematical reaction-diffusion model of ROS-induced ROS release was presented [37]. It demonstrates how ROS release could be realized in short period. However, it is not known how the initial release is triggered and independently regulated from cytochrome c release. We modeled the disposal of ROS in response to TNFα stimulus using the variable 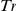 and a function with a narrow peak at 4 hours representing the release of 100 units of ROS. If this burst happens and the amount of ROS cannot anymore be neutralized by a scavenger protein P or fully react with MKPs, remaining ROS persist in the cytoplasm at a high level in our simulations (Figure 6C,D) as shown experimentally (Figure 2A,B). In our experiments the antioxidant BHA is used which is known as potent ROS scavenger. As we could show that BHA completely abolishes the occurrence of ROS in the cell (Figure 2B), we realized its impact with a logical variable set to “1” if BHA is present and set to “0” if BHA is not present.
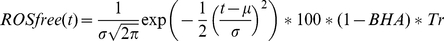
The NF-κB module delivers P mRNA which is translated to the P protein (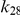). If ROS is released, its amount is buffered by P protein (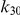). The parameter 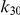 is set to 1 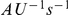 to neutralize ROS after TNFα stimulation fast enough to repress further effects. This scavenging mechanism explains the observation that only the combination of TNFα plus ActD leads to a significant increase in intracellular ROS amounts (Figure 2A). After ActD treatment P mRNA is no longer synthesized and therefore ROS is not scavenged and can display its impact.
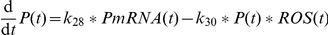
The effect of ROS on the network will now close the loop to the upstream effects of TNFα. As mentioned above, MKPs dephosphorylate pJNK. MKPs themselves can be oxidized and are thereby inactivated by ROS (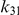) [10]. There is also a backward reaction to this oxidation (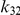).
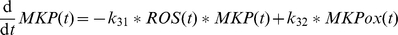


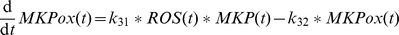
Finally, we give the equation for ROS which are buffered by P (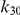) and consumed by the oxidation of MKPs (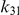).
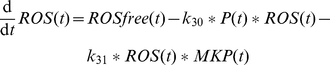
Summarized, ROS can increase the amount of pJNK via two different routes. The oxidation of MKPs via ROS after ActD removes the dephosphorylation mechanisms for pJNK. Thereby the regulative loop consisting of MKPs, pJNK, MKK7 and its phosphatase, is broken. Additionally, our experiments revealed a correlation between ROS and highly increased amounts of unphosphorylated JNK. Via these two mechanisms strong and sustained pJNK activation is established after ROS release in the model reflecting our experimental results (Figure 3B).

### Parameterization of the model in accordance with a complex requirement profile

The initial values of the species from the sensitizing model [20] and from the NF-κB model [21] are maintained. As their absolute amount is not known the initial values of the species DISC, cFLIP, complex0, JNK, proMKK7, Itch and prophosphatase are set to 100% according to [20]. The initial amount of MKP protein is 50% which was set in relation to 100% JNK and ROS, respectively. The initial amount of FADD was set to 200% as it represents a common pool for complex2 and DISC assembly but there is no hint on competitive behavior of these two processes to our knowledge. All other species have initial value zero.

The reaction parameters for the model extension are fitted using a manually iterative approach as proposed in [21] and are listed in Table 1. The goal of the parameter fitting was to fulfill the following qualitative overall constraints in accordance to our experimental results in murine hepatocytes. The model has to show no caspase-3 activation after TNFα, medium caspase-3 activation after Fas ligand and high caspase-3 activation after TNFα/Fas ligand sensitizing as shown in [20] for wildtype hepatocytes. In addition, it was shown that the stronger apoptosis in XIAP knockout mice after Fas ligand is not further enhanced by the sensitizing effect [20]. The experimental results in this study request the model to reproduce high caspase-3 activation in wildtype cells after TNFα plus ActD treatment but not any caspase-3 activation if BHA is added to this experimental setting. Moreover, it was shown that TNFα plus ActD treatment does not induce apoptosis to a remarkable extent in Bid knockout mice [22].

**Table 1:** Parameter values for the extended TNFα-induced apoptosis model.

parameter	value	unit	character of interaction
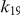	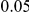	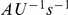	assembly of complex1
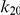	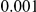	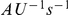	assembly of complex2
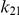	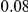	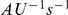	activation of caspase-8 at complex2
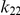	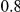	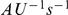	activation of caspase-8 at the DISC
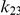	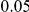	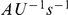	activation of MKK7 by complex1
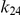	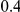	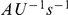	phosphorylation of JNK by MKK7
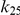	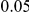	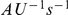	activation of prophosphatase by pJNK
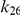	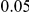	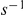	decay of active phosphatase
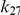	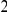	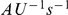	dephosphorylation of MKK7 by phosphatase
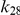	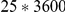	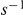	translation of protein P
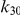	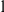	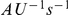	absorption of ROS by protein P
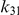	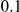	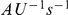	oxidation of MKPs by ROS
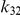	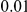	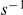	recovering of oxidized MKPs
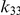	2	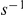	linear increase of the amount of JNK in relation to ROS
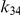	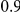	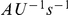	dephosphorylation of pJNK by MKPs
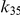	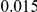	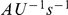	phosphorylation of itch by pJNK in presence of CHX
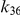	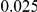	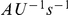	phosphorylation of itchP by pJNK
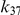	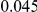	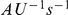	phosphorylation of itchPP by pJNK
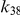	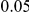	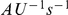	degradation of cFLIP by itchPPP
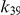	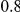	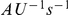	inhibition of complex2 by cFLIP
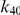	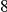	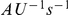	inhibition of DISC by cFLIP
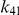	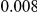	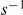	decay of complex c2FLIP
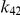	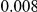	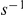	decay of complex DcFLIP
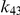	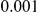	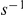	turnover of complex0
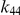	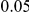	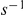	decay of complex1
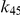	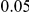	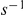	decay of complex2
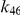	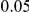	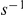	decay of the DISC
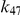	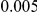	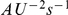	assembly of the DISC

Parameter identifiers 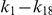 have been used in [20] and are listed in the supplementary information (Model S1). As this is a qualitative model the parameter values are not identical with kinetic constants at the level of elementary reactions and are declared in artificial units (AU).

To conform to these requirements the different routes to caspase-3 activation need to be in a precise balance which is established to a large extent by the parameterization of the equations in connection with the species cFLIP. The parameter 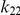 for the activation of caspase-8 at the DISC was set to 0.8 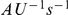. As it was shown that cFLIP is bound preferentially to caspase-8 at the DISC [38] the parameter 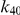 for the inhibition of caspase-8 activation at the DISC by cFLIP is defined to be 8 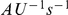 thereby also guaranteeing the apoptotic threshold realized by cFLIP [39]. The same factor is assumed for the competition of caspase-8 and cFLIP at complex2 (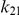, 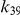) but the activation of caspase-8 in the model is ten times lower at complex2 than at the DISC. This parameterization guarantees that Fas ligand is activating sufficient caspase-3 in the model to overcome the XIAP buffer but not inducing cytochrome c release and TNFα does not lead to significant caspase-3 activation. However, TNFα activates sufficient caspase-8 via complex2 to cleave some Bid molecules. The increased levels of pJNK after TNFα plus ActD treatment phosphorylate the complete pool of Bim and thereby exhaust the Bcl2 buffer in the model. However, TNFα plus ActD treatment does not lead to enhanced apoptosis in hepatocytes from Bid knockout mice [22]. Therefore, an equivalent amount of Bim and Bcl2 is modeled and an additional apoptotic impact of TNFα via caspase-8 and tBid is necessary.

A critical point is also the regulation of JNK phosphorylation after TNFα and ActD. The according parameters are set to be 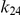 = 0.4 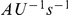 for the phosphorylation of JNK by MKK7 and 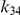 = 0.9 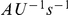 for the even faster dephosphorylation of pJNK by MKPs. These parameters allow for rapid adjustment of the balance between JNK and pJNK in reaction to signals but also guarantee a quick termination of the pJNK signal. Thereby the measurements showing pJNK appearing in a first rapid and very short peak which is terminated abruptly can be reproduced. The termination of the first peak needs active MKPs and follows the downregulation of MKK7 via the according active phosphatase induced by pJNK itself. However, some MKK7 proteins still need to be active at later time points to induce the second activation phase of JNK. Therefore, the balance between prophosphatase, phosphatase, complex0, complex1, complex2 and MKK7 is delicate. The second JNK phosphorylation is triggered by ROS oxidizing MKPs efficiently (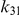 = 0.1 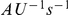) which in turn recover slowly (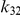 = 0.01 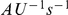). Additionally, ROS is related to a strongly increased amount of JNK. Overall ten different species are now involved in JNK regulation in the model in contrast to a single species in the sensitizing model [20].

In Supplementary Information S1, we provide local sensitivity coefficients of pJNK, activated Bax/Bak and activated C3 with respect to the model parameters after stimulation with TNF. These coefficients show to which extent the single parameters push the system to survival or apoptosis. We further provide a robustness analysis, where we test to which degree single parameters can be disturbed without changing the apoptosis/survival pattern in the wildtype after different stimulations. Parameters of processes that robustly lead to apoptosis (e.g. the formation of DISC complex 2) can be varied by four orders of magnitude without influencing the decision of survival versus apoptosis. Parameters describing important control points at the complex 1 and 2, the Bim/Bid module, the phosphorylation of JNK and the the NF-κB module show a much less robust behaviour.

### The modeled interplay of species successfully reproduces the measurement data

Figure 6 shows simulation results for TNFα stimulation (A–C), for TNFα plus ActD stimulation (D–F) and simulations of the TNFα/Fas ligand sensitizing effect (G–I). After TNFα stimulation there is only one peak of JNK activation at around 30 min which is terminated by MKPs in between 60 min (6C). This activation is not sufficient to phosphorylate a significant amount of Bim (6B). ROS are released after four hours (6C); however, they are neutralized by protein P which was induced by TNFα activating the NF-κB module (6A). In contrast, high ROS levels remain in the cell (6D) and oxidize MKPs (6F) after concomitant treatment with TNFα and ActD because P is not produced. ROS induce a strong increase in the amount of JNK (6F). For these two reasons, a second activation of JNK occurs which is not only longer lasting but also at a far higher level (6F). In addition, there is weak activation of caspase-8 at complex2 (6E). The produced protein forms tBid and pBim lead to exhaustion of the Bcl2 buffer and finally to cytochrome c release mediated by active Bax/Bak (6E). Thereby caspase-3 is activated to the maximally possible amount in the model which is restricted in wildtype cells by the presence of XIAP. Finally, the model is used to simulate the sensitizing effect. Equivalently to the experimental conditions, TNFα is given as model input from time point zero and after 12 hours Fas stimulus is added. For the first 12 hours the simulation is identical with TNFα only. After Fas ligand is added at 12 hours there is strong activation of caspase-8 at the DISC and also tBid is produced which finally leads to cytochrome c release (6H). Caspase-3 is activated in this scenario by active caspase-8 directly as well as by cytochrome c. However, caspase-3 is not activated to a higher extent as after TNFα plus ActD treatment. The reason is that cytochrome c is released in both scenarios and its strong impact covers the additional direct caspase-3 activation by caspase-8.

The simulations after concomitant treatment with TNFα, ActD and BHA are shown in Figure S2. There is no ROS release due to BHA, accordingly MKPs remain and a second pJNK increase is missing in the model which is in agreement with our experimental results (Figure 3C). Consequently, caspase-3 is not activated as it is shown experimentally in Figure 2C.

Simulations of the model after treatment with TNFα plus cycloheximide are shown in Figure 7. The amount of JNK and pJNK is increased in this setting as described above for TNFα plus ActD. In the presence of cycloheximide pJNK additionally phosphorylates Itch (7A). The threefold phosphorylated form of Itch degrades cFLIP and thereby a high amount of caspase-8 is activated in this scenario (7C). However, the model does not predict a difference in caspase-3 activity whether TNFα is combined with ActD or with cycloheximide as again the scenario is determined by 100% cytochrome c release.

## Discussion

Using ordinary differential equation (ODE) models is a standard approach in systems biology as it allows describing dynamic behavior over time. A drawback of this approach is however the dependency on kinetic parameters for the simulation of an ODE model. For biological interactions, these parameters usually cannot be directly measured and need to be identified from measurement data. These data are most often not quantitative in absolute units and is afflicted with uncertainty. In addition, many model parameters are in fact not elementary biochemical parameters but subsume several processes which are not modelled in detail. This might be one of the reasons that ODE models for biological systems are rarely merged into larger models although a variety of signalling pathway models is already available. There are supporting approaches to technically merge mathematical models [40]. However, model integration is a complex task as different mathematical models usually do not adhere to a common modeling standard.

We extended an existing model of TNFα and FasL sensitizing by adding the regulation of pJNK and its impact on the signaling network. Thereby ROS signaling and a published NF-κB model were integrated in the framework. The presented mathematical model is based on biological experiments and on literature. Overall the model comprises 53 different species (thereof 16 in the NF-κB module) and 74 reaction parameters.

The presented extended model still reproduces the sensitizing effect which was a presupposed quality requirement. During the integration of the sensitizing model into the far bigger framework we present here, overall seven parameters from the originally published model have been changed. JNK is now activated in a peak-like fashion which is in accordance with our measurement results (Fig. 3). Due to the adjusted parameterization of the model Bax/Bak is now not activated at all after a sole TNFα or Fas ligand stimulation. As a certain level of active Bax/Bak can finally lead to cytochrome c release, the more well-defined difference between not any and high Bax/Bak activation is equivalent with a more stable type I apoptosis in the model. Overall the reproduction of the sensitizing effect is not only maintained but even improved.

Moreover, the presented expanded model for TNFα-induced apoptosis now also reproduces apoptosis after combined TNFα and ActD treatment as well as the effect of BHA. The qualitative interactions in the network are based on literature data and the presented measurement data for murine hepatocytes which clearly document the modeled functional mechanisms.

In this work, a special focus lies on the intention to analyze several pathways that have been associated with TNFα-induced apoptosis but measured rather separately. Although knowledge exists on the regulation and implication of JNK [41], the crosstalk of JNK and NF-κB [8] and the relevance of ROS accumulation and JNK prolongation [32], the overall analyses of these pathways in one cell type is still rare. Experimental data have been reported for each single of these processes in several cell types but the interplay of JNK, NF-κB and ROS has been shown here in more detail in primary murine hepatocytes. In this context, it is also known that TNFα such as other death receptor ligands can trigger either type I or type II apoptosis pathway [42], and that very different regulation signals are involved in both. Having demonstrated that TNFα activates type II apoptosis signaling, this pathway could in this work be further linked to the JNK, ROS and NF-κB pathways in hepatocytes.

In particular, it could be shown that TNFα plus ActD induce apoptosis by ROS-mediated sustained activation of JNK. Furthermore, the inactivation of NF-κB-mediated survival gene transcription was shown to be critically involved as it has been reported before [17], [43]. Because ROS is supposed to act upstream of JNK, inhibition of ROS accumulation by NF-κB is assumed to be one major regulatory step [44]. ROS can be neutralized by superoxide dismutases (SODs). Especially MnSOD has been shown to be upregulated by NF-κB and to suppress TNFα-mediated ROS accumulation and cell death in MEFs and cancer cells [10], [45]. However, we could not confirm any MnSOD upregulation in response to TNFα treatment nor the reduction of MnSOD protein in response to TNFα plus ActD treatment. Thus, other NF-κB-dependent mechanisms seem to control the ROS-mediated activation of JNK in primary murine hepatocytes. Interestingly, NF-κB induces the expression of ferritin heavy chain (FHC), which binds and stores iron ions which reduce ROS levels and inhibit prolonged JNK activation [28]. Several other NF-κB target genes have been associated with inhibition of the JNK signal, namely Gadd45β, which inhibits JNK by blocking the catalytic activity of MKK7 [27], XIAP [43] and A20 [46]. Taken together, the NF-κB-mediated control of ROS and JNK seems to be composed of several regulatory proteins and has to be determined for each cell type specifically.

In this work we report the unexpected finding that ROS upregulate the levels of JNK protein and that this upregulation might be the reason for an increase in phosphorylated JNK. Moreover, first experiments in primary murine hepatocytes suggest that this ROS-mediated JNK upregulation might be controlled by JNK itself because inhibition of JNK activity abrogates the reported increase in JNK protein levels (data not shown). However, this regulatory step should be investigated in more detail in order to understand the exact mechanisms involved.

Astonishingly, one of the major regulators of caspase-8 activation, cFLIP, which has been shown to be degraded in TNFα-mediated apoptosis [13] remained unchanged upon TNFα plus ActD treatment in our experiments. However, Chang et al. demonstrated cFLIP degradation in response to TNFα plus cycloheximide which we could reproduce in murine hepatocytes (data not shown). Accordingly, it can be suggested that the apoptosis pathway induced by TNFα plus ActD differs from the mechanism via the JNK and Itch-mediated cFLIP degradation and predominantly caspase-8 activation induced by TNFα plus cycloheximide as shown by Chang et al [13]. Interestingly, TNFα has recently been reported to induce two distinct caspase-8 dependent apoptosis pathways [47]. In this study, Wang et al. demonstrated that autodegradation of cIAPs by smac mimetics triggers the formation of a new caspase-8 activating complex while TNFα and cycloheximide induce caspase-8 activation via cFLIP degradation and complex II formation. Thus, it could be speculated that this TNFα-Smac mimetic-mediated apoptosis pathway may be similar to the one observed in response to TNFα plus ActD treatment.

In our model we assume TNFα plus ActD to predominantly act by the ROS-mediated strong and sustained activation of JNK which results in the generation of pBim [20], [48]. Large amounts of pBim cooperate with tBid to finally activate the mitochondrial pathway and result in cytochrome c release. This finding has been shown to be even true in-vivo in the case of fatal hepatitis and seems to be of general relevance in the control of hepatocyte apoptosis during liver disease [16], [49].

The experimental approach together with the extended model provides a deep and more complete insight in TNF signaling in murine hepatocytes. There are further participating interactions that are currently not included in the model. For example, XIAP and MKPs are known NF-κB target genes, too [43]. This could exert at least a quantitative influence in the analyzed setting. However, our mathematical model shows that the modeled network is able to reproduce the measurement data.

It is sometimes not so difficult to model a certain qualitative behavior with a very simple, reduced model while neglecting the connections and crosstalks of the modeled species in the signaling framework of the cell. In the case of NF-κB a lot of effort has been put in mathematical modeling in order to understand the dynamics of this pathway (reviewed in [50]). However, many models focus on isolated steps of the signaling such as e.g. IKK dephosphorylation [51]. The TNF receptor has been employed to trigger mathematical NF-κB models in these studies [52], [53] but no other signaling pathways have been included. Cho *et al.* take the amount of FADD complex as indicator for apoptosis in their NF-κB model but without modeling e.g. caspases [54]. There are several models dealing with caspase activation and inhibition in type I apoptosis [55] or type II apoptosis [56]–[60]. However, these models also in turn do not comprehend NF-κB or other crosstalking signaling pathways. If any receptor species is used these models also include either TNFR [60] or Fas/CD95 [57], [59]. Examples for more comprehensive models include a model of TRAIL-induced apoptosis with detailed mitochondrial module [61], a model of CD95L-induced apoptosis with highly detailed caspase module [39] and a model of TNFα-induced apoptosis with a simple NF-κB module [62]. All these models provide valuable insights into their respective signaling pathway. However, they originally cannot reflect behavior patterns which emerge from the crosstalk between different pathways as we present them in this study.

Systems biology just started to pass on from dynamic modeling of single pathways to dynamic modeling of comprehensive and intensively crosslinked networks. More information can be gained by adding further levels of detail in existing models [50], [63], [64]. Here, we provide a first framework that helps to explain the complex interactions between TNFα signaling, Fas signaling and transcriptional activity. We intend to give a motivating example for integration of several dynamical mathematical models into a holistic model describing complex crosstalk. The presented model can be used for integration of even more models in the context of the apoptosis/survival switch to finally improve the analysis of all involved pathways and the understanding of the holistic network. As especially the Bcl-2 family and NF-κB are central network hubs in the cell there are many signaling pathways which have connecting points with the model presented here. They could all be mathematically modeled and integrated to analyze their impact on apoptosis but also the influence of apoptotic signaling to them. The extensive cross-linking of dependencies can thereby lead towards a stepwise improvement of the network model as every additional player restricts degrees of freedom for others.

## Supporting Information

Figure S1 **cFLIP and MnSOD mRNA are moderately upregulated by TNFα while ActD treatment abrogates their expression.** Primary murine hepatocytes were treated with TNFα (25 ng/ml) with or without ActD (0.4 µg/ml) for the indicated times and cFLIP (A) and MnSOD (B) mRNA levels determined by qRT-PCR. Means of at least three independent experiments ± s.d. are shown.
(TIF)

Figure S2 **Simulation results of the TNFα-induced apoptosis model after TNFα, ActD and BHA.** (A–C) Simulation results for pivotal species of the TNFα-induced apoptosis model over 10 hours after concomitant stimulation with TNFα, ActD and BHA. The species are shown in separate panels for clarity as indicated in the legend.
(TIF)

Model S1 **Additional information concerning the model.**
(PDF)

Model S2 ***Mathematica*** **file (see** http://www.wolfram.com/ **) containing the model.**
(NB) Click here for additional data file.

Supplementary Information S1 **Experimental procedures.**
(DOC)
