# Supplementary figures and images for: Modeling the TNFα-Induced Apoptosis Pathway in Hepatocytes
Source: PLoS One. 2011 Apr 20;6(4):e18646. doi: 10.1371/journal.pone.0018646 (PMC3080376; doi:10.1371/journal.pone.0018646)

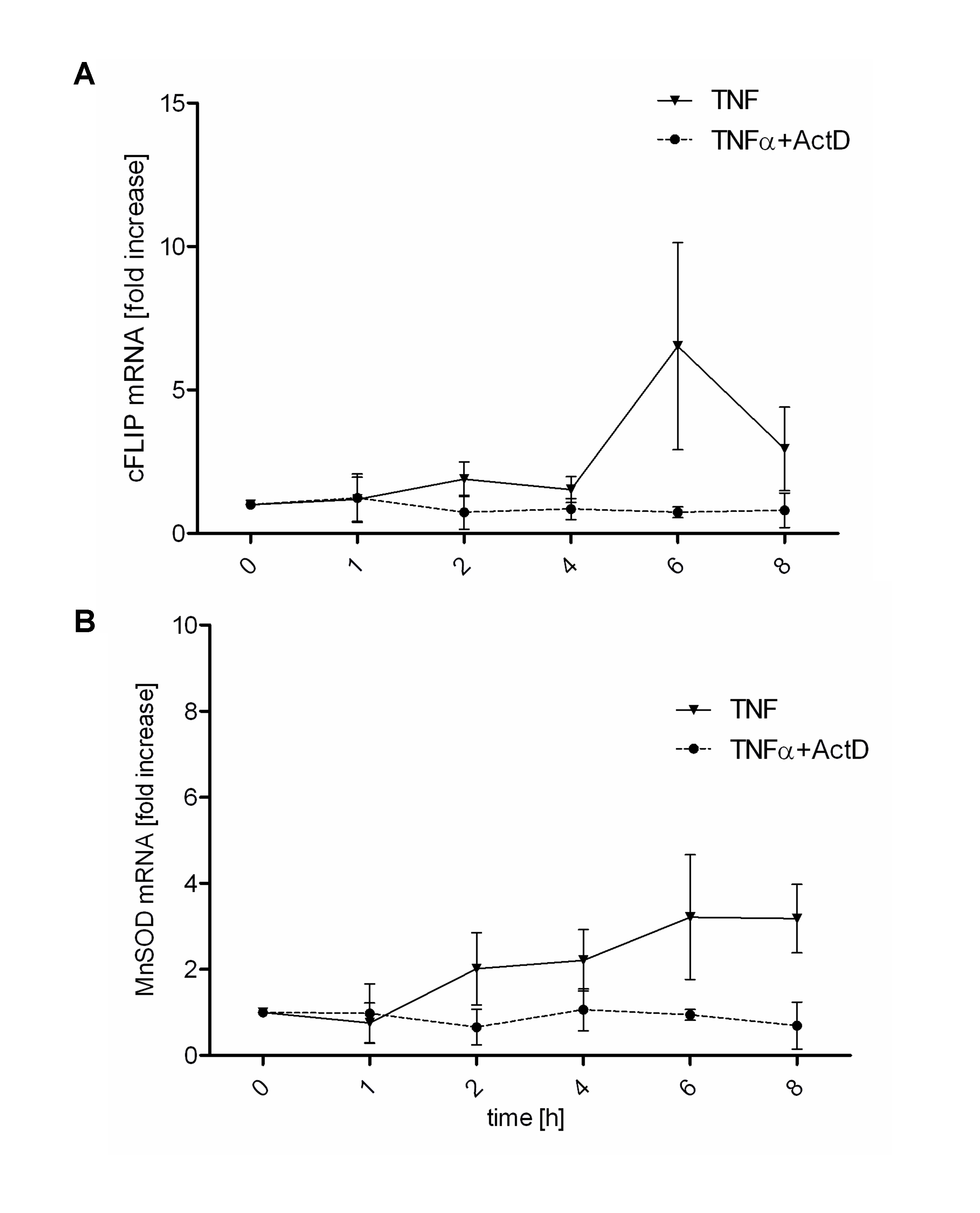

Supplement: Figure S1 — cFLIP and MnSOD mRNA are moderately upregulated by TNFα while ActD treatment abrogates their expression. Primary murine hepatocytes were treated with TNFα (25 ng/ml) with or without ActD (0.4 µg/ml) for the indicated times and cFLIP (A) and MnSOD (B) mRNA levels determined by qRT-PCR. Means of at least three independent experiments ± s.d. are shown. (TIF) [file pone.0018646.s001.tif]

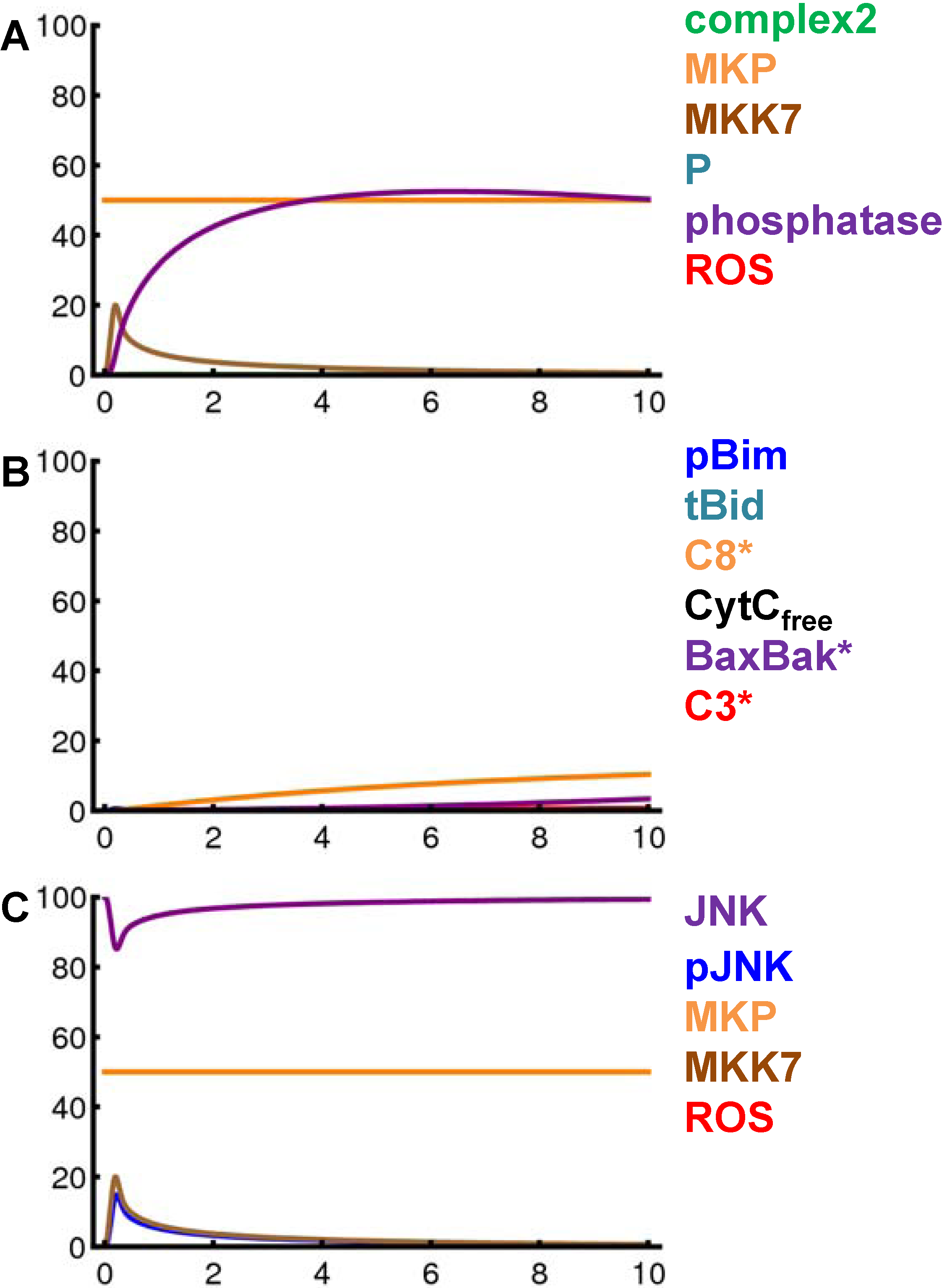

Supplement: Figure S2 — Simulation results of the TNFα-induced apoptosis model after TNFα, ActD and BHA. (A–C) Simulation results for pivotal species of the TNFα-induced apoptosis model over 10 hours after concomitant stimulation with TNFα, ActD and BHA. The species are shown in separate panels for clarity as indicated in the legend. (TIF) [file pone.0018646.s002.tif]
