# Supplementary material for: Modeling the TNFα-Induced Apoptosis Pathway in Hepatocytes
Source: PLoS One. 2011 Apr 20;6(4):e18646. doi: 10.1371/journal.pone.0018646 (PMC3080376; doi:10.1371/journal.pone.0018646)
Supplement: Model S1 — Additional information concerning the model. (PDF) [file pone.0018646.s003.pdf]

Supplementary Material to

"Modeling the TNFα-induced Apoptosis Pathway in Hepatocytes"

Comparison with the TNFα-induced apoptosis model with the mathematical model of the sensitizing effect (Schmich et al., 2011), sensitivity and robustness analysis.

Equations and scheme of the sensitizing model taken from Schmich et al., 2010:

$$\frac{d\mathrm{TNF}[t]}{dt} = 0$$
$$\frac{d\mathrm{JNK}[t]}{dt} = -v_1$$
$$\frac{d\mathrm{pJNK}[t]}{dt} = v_1 - v_2$$
$$\frac{d\mathrm{Bim}[t]}{dt} = -v_3$$
$$\frac{d\mathrm{pBim}[t]}{dt} = v_3 - v_4 - v_5$$
$$\frac{d\mathrm{C8}[t]}{dt} = -v_7$$
$$\frac{d\mathrm{C8^*}[t]}{dt} = v_7 - v_8$$
$$\frac{d\mathrm{C3}[t]}{dt} = -v_{14} - v_{15} - v_{16}$$
$$\frac{d\mathrm{C3^*}[t]}{dt} = v_{14} + v_{15} + v_{16} - v_{17} - v_{18}$$
$$\frac{d\mathrm{Bid}[t]}{dt} = -v_9$$
$$\frac{d\mathrm{tBid}[t]}{dt} = v_9 - v_{10} - v_{11}$$
$$\frac{d\mathrm{BaxBak}[t]}{dt} = -v_6 - v_{12} + v_{13}$$
$$\frac{d\mathrm{BaxBak^*}[t]}{dt} = v_6 + v_{12} - v_{13}$$
$$\frac{d\mathrm{XIAP}[t]}{dt} = -v_{18}$$
$$\frac{d\mathrm{Bcl2}[t]}{dt} = -v_5 - v_{11}$$

$$\mathrm{Fas}[t] = UnitStep[t - \alpha] * 100$$

$$\mathrm{CytC_{free}}[t] = UnitStep[\mathrm{BaxBak^*}[t] - 90] * 100$$

$UnitStep[x]$ represents a function equal to 0 for $x < 0$ and 1 for $x \geq 0$. In case of Fas stimulation after 12 hours α is set to 12 and in scenarios without Fas stimulation α is set to $(t_{\mathrm{maximal}} + 1)$.

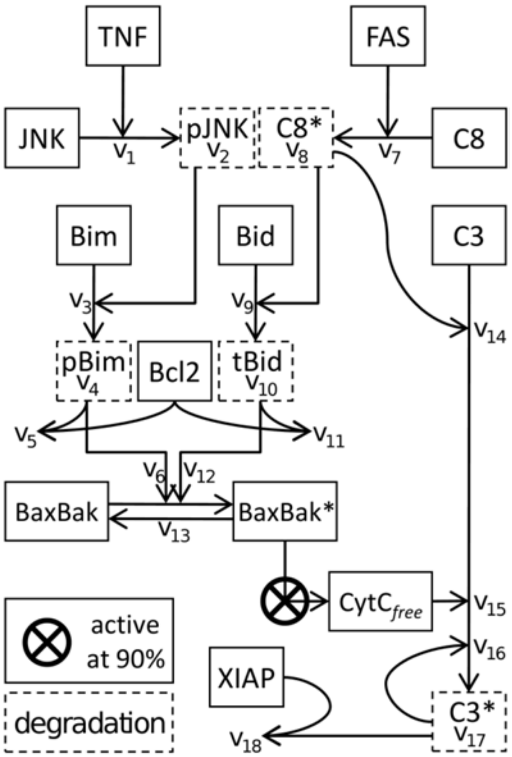

$$v_1 = k_1 * \mathrm{TNF}[t] * \mathrm{JNK}[t]$$
$$v_2 = k_2 * \mathrm{pJNK}[t]$$
$$v_3 = k_3 * \mathrm{pJNK}[t] * \mathrm{Bim}[t]$$
$$v_4 = k_4 * \mathrm{pBim}[t]$$
$$v_5 = k_5 * \mathrm{pBim}[t] * \mathrm{Bcl2}[t]$$
$$v_6 = k_6 * \mathrm{pBim}[t] * \mathrm{BaxBak}[t]$$
$$v_7 = k_7 * \mathrm{Fas}[t] * \mathrm{C8}[t]$$
$$v_8 = k_8 * \mathrm{C8^*}[t]$$
$$v_9 = k_9 * \mathrm{C8^*}[t] * \mathrm{Bid}[t]$$
$$v_{10} = k_{10} * \mathrm{tBid}[t]$$
$$v_{11} = k_{11} * \mathrm{tBid}[t] * \mathrm{Bcl2}[t]$$
$$v_{12} = k_{12} * \mathrm{tBid}[t] * \mathrm{BaxBak}[t]$$
$$v_{13} = k_{13} * \mathrm{BaxBak^*}[t]$$
$$v_{14} = k_{14} * \mathrm{C3}[t] * \mathrm{C8^*}[t]$$
$$v_{15} = k_{15} * \mathrm{C3}[t] * \mathrm{CytC_{free}}[t]$$
$$v_{16} = k_{16} * \mathrm{C3}[t] * \mathrm{C3^*}[t]$$
$$v_{17} = k_{17} * \mathrm{C3^*}[t]$$
$$v_{18} = k_{18} * \mathrm{C3^*}[t] * \mathrm{XIAP}[t]$$

Original parameter and initial values used for the sensitizing model in Schmich et al., 2010 and their adaption for the TNFα-induced apoptosis model:

Parameter values:

parameter	description	original value	new value	comment
k_1	activation of JNK by TNF	0.08	-	dropped out
k_2	degradation of pJNK	0.9	-	dropped out
k_3	phosphorylation of Bim by pJNK	0.04	0.04	not changed
k_4	degradation of pBim	0.001	0.001	not changed
k_5	binding of Bcl2 and pBim	0.1	1	fitted
k_6	activation of BaxBak by pBim	0.005	0.005	not changed
k_7	activation of C8 by Fas	0.01	-	dropped out
k_8	degradation of active C8	0.01	0.01	not changed
k_9	cleavage of Bid by C8	0.001	0.002	equalized with k_{14}
k_{10}	degradation of C8	0.001	0.001	not changed
k_{11}	binding of Bcl2 and tBid	0.1	1	fitted
k_{12}	activation of BaxBak by tBid	0.05	0.1	fitted
k_{13}	inactivation of active BaxBak	0.0001	0.0001	not changed
k_{14}	cleavage of C3 by C8	0.002	0.002	not changed
k_{15}	activation of C3 by cytochrome c	0.05	0.05	not changed
k_{16}	autoactivation of C3	0.007	0.007	not changed
k_{17}	degradation of C3	0.01	0.01	not changed
k_{18}	binding of C3 and XIAP	0.05	0.05	not changed

As this is a qualitative model the parameter values are not identical with kinetic constants at the level of elementary reactions.

Initial values: The initial values for all species included in the sensitizing model from Schmich et al., 2010 are not altered during adaption but remain:

TNF[0] = 100 or 0 (input), JNK[0] = 100, pJNK[0] = 0, Bim[0] = 100, pBim[0] = 0, C8[0] = 100, C8*[0] = 0, C3[0] = 100, C3*[0] = 0, Bid[0] = 100 or 0 (wildtype/knockout), tBid[0] = 0, BaxBak[0] = 100, BaxBak *[0] = 0, XIAP[0] = 80 or 0 (wildtype/knockout), Bcl2[0] = 100,

Start values for all species pools have been set to 100%. Solely XIAP[0] is set to 80% to reflect the measured relation between XIAP and caspase-3. It was found that at most about 20% caspase-3 is active in wildtype hepatocytes in comparison with XIAP knockout hepatocytes (Schmich et al., 2010).

Sensitivity Analysis

In order to analyse the influence of the parameter values on the model results we conducted a sensitivity analysis for the enhanced sensitizing model presented in the manuscript. We computed local sensitivity coefficients describing the reaction of the model for a 1% change of the parameters. Further we conducted a robustness analysis to analyse which parameters are crucial in model for the apoptosis decision.

1) Local Sensitivity Coefficients

Local sensitivity coefficients show how the model results change if parameter values are slightly varied. The sensitivity coefficients allow distinguishing parameters with a high influence on the model variables from those with a low influence.

We computed simulation results with the nominal parameter set of the model. Then, we successively increased each single parameter by 1% and computed simulation results for the perturbed parameter values. The scaled sensitivity coefficients are the relative changes of the simulation divided by the relative change of the parameter:

$$S_{x_j, p_i} = \frac{x_j(p_1, \dots p_i + \Delta p_i, \dots p_m, t) - x_j(p_1, \dots p_i, \dots p_m, t)}{\Delta p_i} \cdot \frac{p_i}{x_j(p_1, \dots p_i, \dots p_m, t)}$$

where $x_j(p_1, \dots p_i, \dots p_m, t)$ is the simulation result for the jth state variable for the given parameters p_i at time t. We use $\Delta p_i = 1.01 \cdot p_i$, i.e. a 1% change of the parameter value. The time-variant sensitivity coefficients indicate by how many percent the model variables change if the parameter values are increased by 1%.

Figures 1, 2 and 3 show the results of the sensitivity analysis for pJNK, activated C3 and activated BaxBak after stimulation of the model with TNF. We chose these variables because they correspond to central points in the model. The scaled sensitivity coefficients at time zero cannot be computed directly because the initial conditions for pJNK, activated C3 and activated BaxBak are zero and in the above formula a quotient of *0/0* occurs. However, finite limit values exist.

After stimulation with TNF, the cells survive because the survival signalling is stronger than apoptosis signalling. The sensitivity coefficients for activated C3 and BaxBak show to which extent the parameters push the system to survival or apoptosis. These sensitivity values may be helpful if the model is further extended and adapted to further scenarios.

2) Robustness Analysis

The local analysis of the previous section reveals the sensitivity of the system towards small parameter changes. However, it is not suited to analyse at which parameter values the transition from survival to apoptosis occurs.
Here, we study by what factors the value of a parameter can vary before the system output is changed qualitatively. The results of such an analysis are a measure for the robustness of the model against parameter variations.

For the following analysis we only consider the amount of active caspase 3. We consider the model to predict apoptosis if the amount of active caspase 3 is above a certain threshold. This threshold is set to a value of 5 in the model units. We consider a set of scenarios (TNF, FAS, TNF + Fas (12h), TNF + actD, TNF + actD + BHA, TNF + Fas + actD, TNF (CHX), FAS (CHX), TNF + Fas (12h) (CHX), TNF + actD (CHX), TNF + actD + BHA (CHX), TNF + Fas + actD (CHX)) and compute if the model predicts apoptosis or survival within 20h. Now we successively vary each single parameter by a factor of 10^n, where n varies from -2 to 2 in 0.1 steps. Thus, we scan four orders of magnitude. A white box in Figure 4 indicates that the perturbed parameter sets leads to the same apoptosis/survival pattern for all scenarios as the nominal parameter set. A black box indicates that at least one scenario leads to a different outcome.

This analysis shows that several parameters can be varied by four orders of magnitude without influencing the decision of survival versus apoptosis. These parameters describe processes that robustly lead to apoptosis (e.g. the formation of DISC complex 2). Many parameters are subject to strong one-sided limitations, i.e. they are restricted by an upper or lower bound (e.g. k_3, k_9, k_{10}, k_{11}, k_{12}, k_{21}, k_{23}, k_{24}, k_{25}, k_{27}, k_{28}, k_{34}, k_{39} and k_{44}). These parameters correspond to important control points at the complex 1 and 2, the Bim/Bid module, the phosphorylation of JNK and the Nf-κB module. These parameters need special focus when extending the model further because their precise values are decisive for the survival/apoptosis switch.

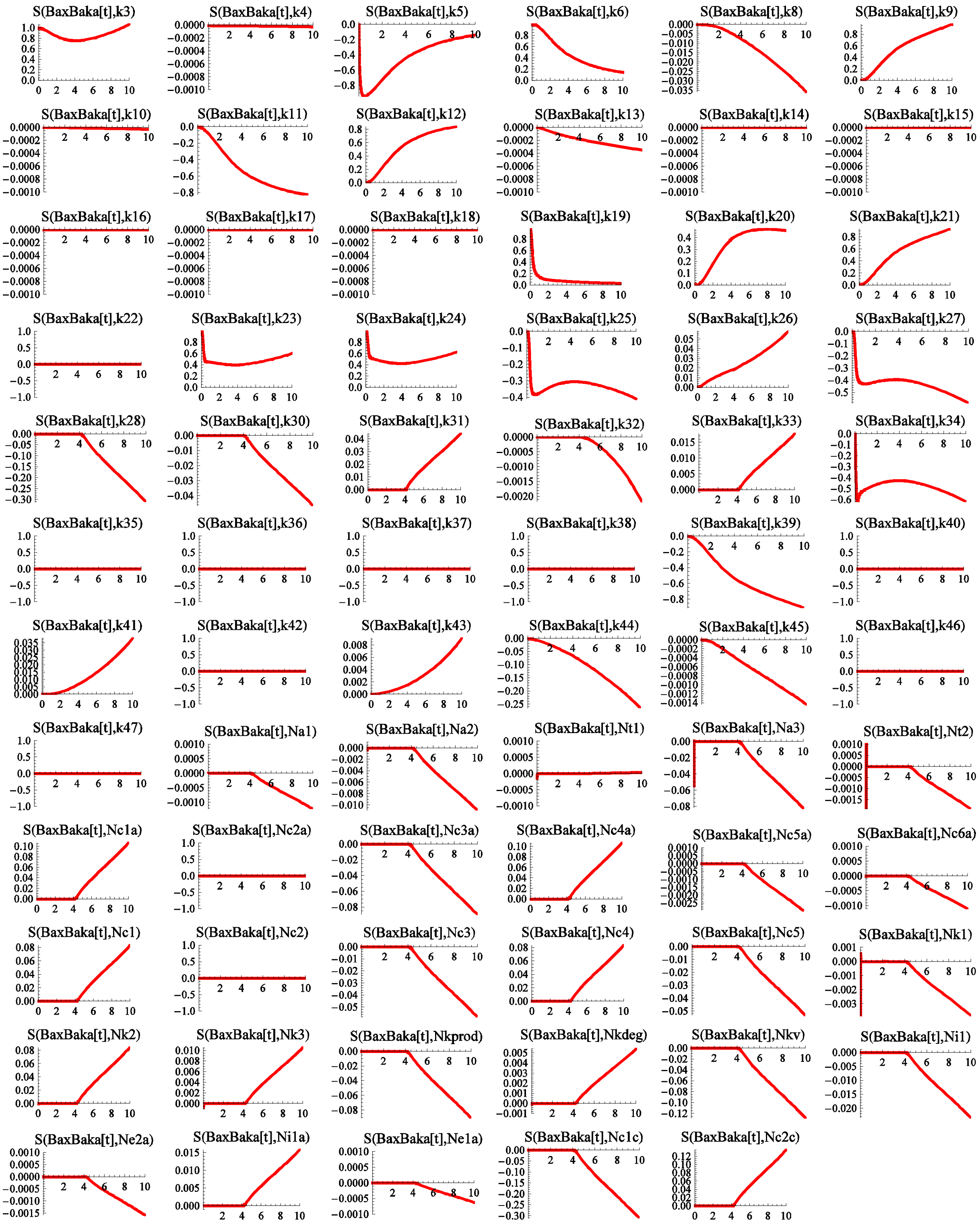

Figure 1. Scaled sensitivity coefficients for activated BaxBak after TNF stimulus.

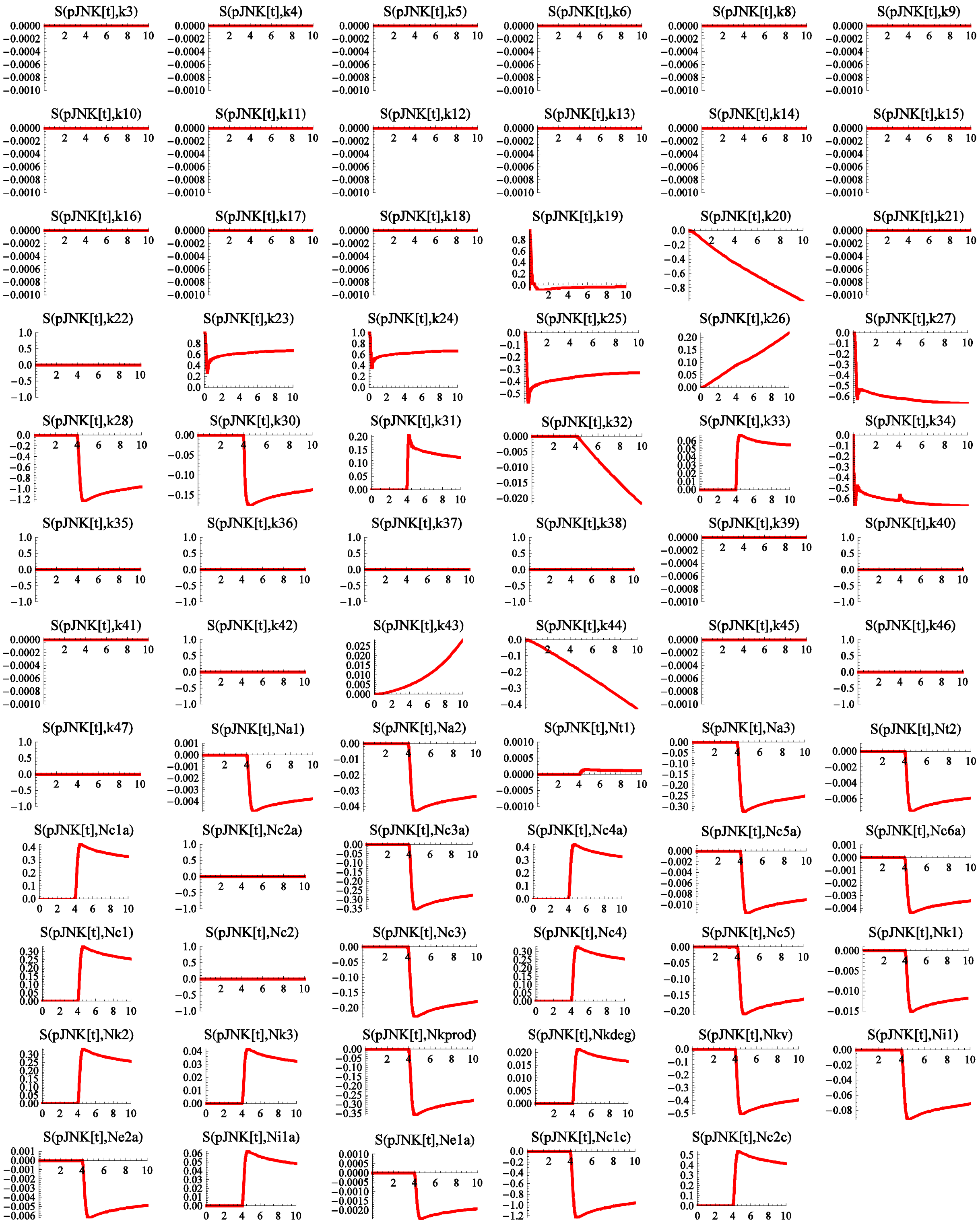

Figure 2. Scaled sensitivity coefficients for pJNK after TNF stimulus.

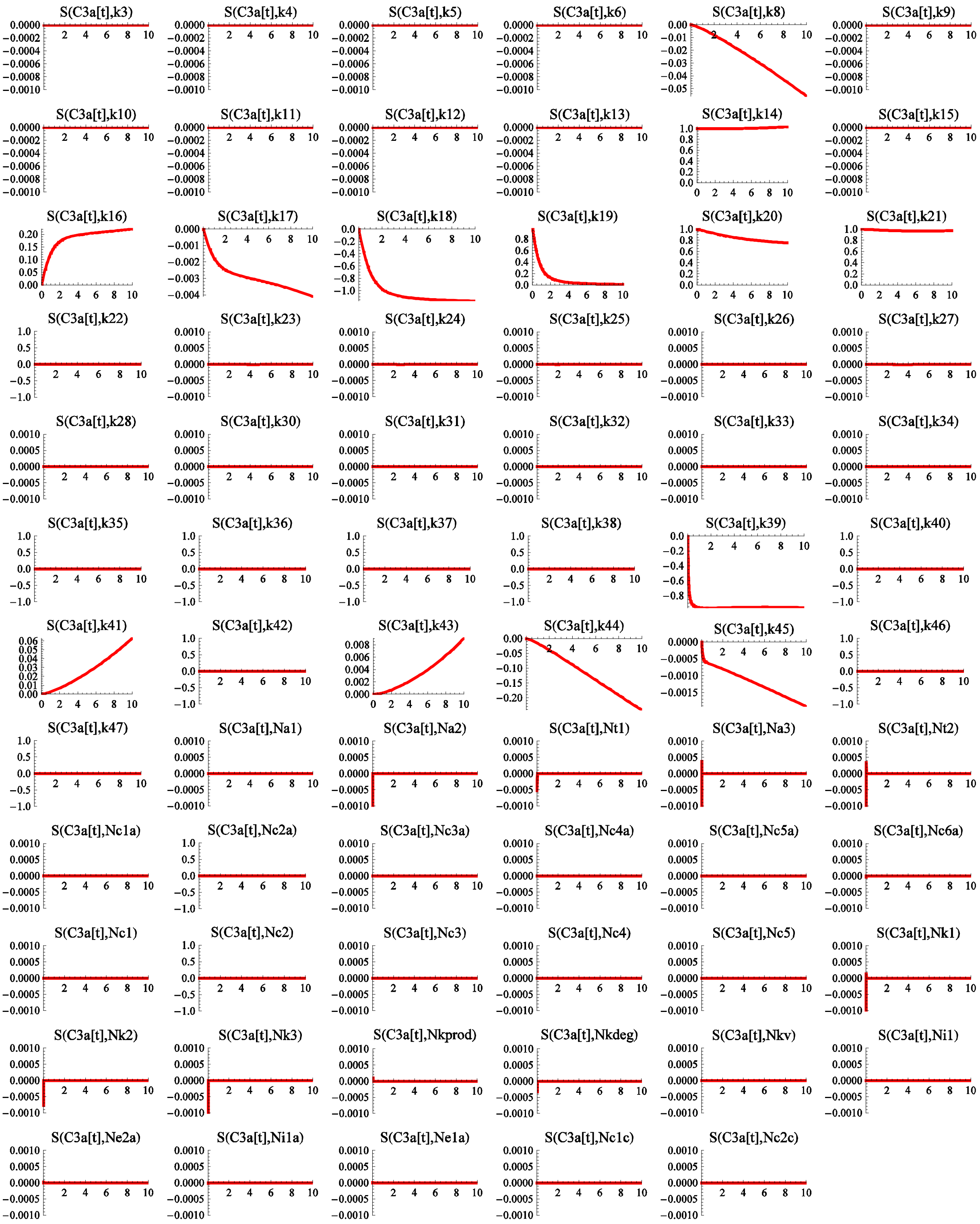

Figure 3. Scaled sensitivity coefficients for activated caspase 3 after TNF stimulus.

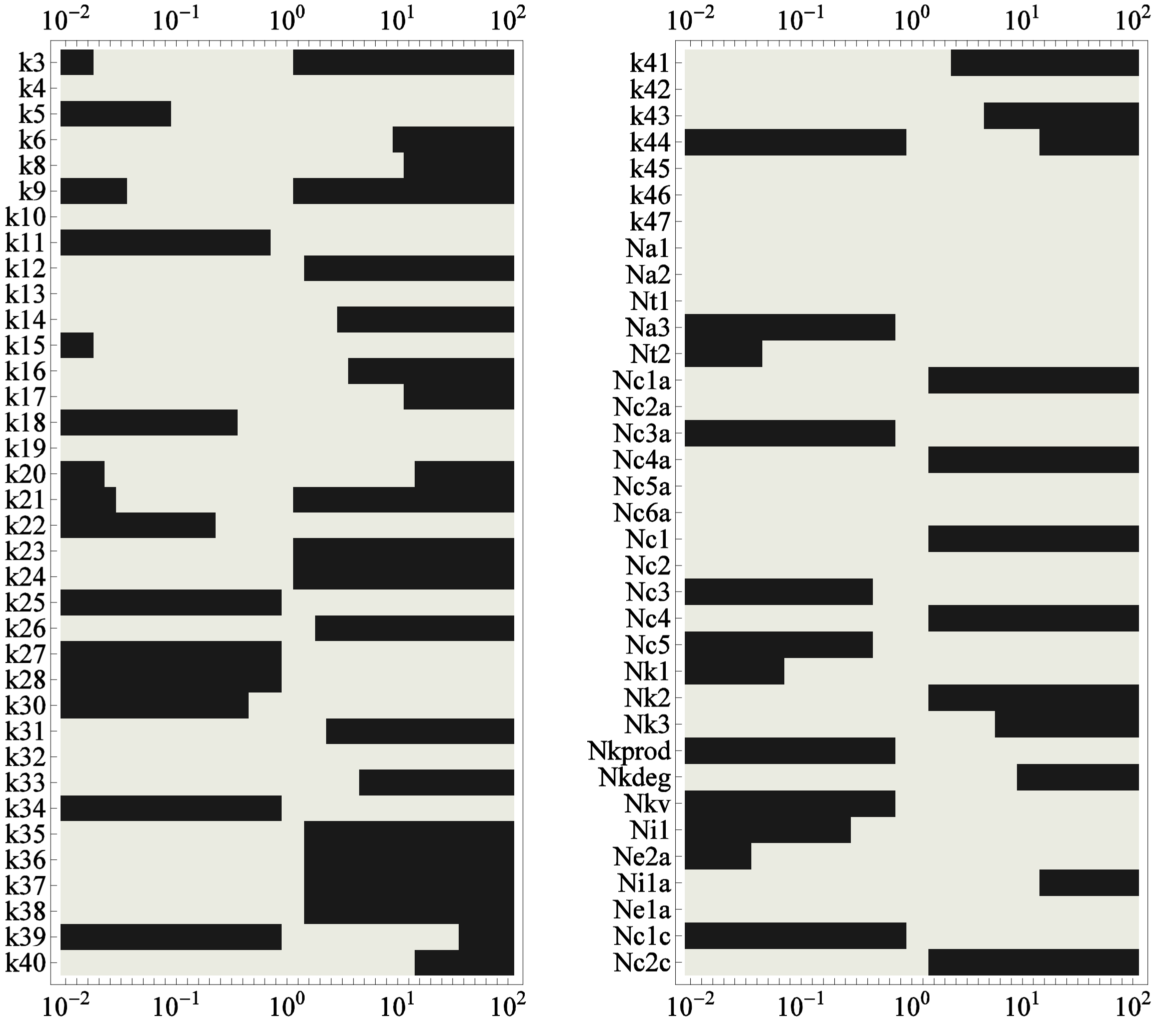

Figure 4. Robustness of the model against parameter changes. The parameters on the ordinate were changed by the factor shown on the abscissa. A white box means that the apoptosis/survival pattern for all considered scenarios is equal to the nominal apoptosis/survival pattern. A black box indicates that at least one difference occurred.
