# Supplementary material for: Modeling the TNFα-Induced Apoptosis Pathway in Hepatocytes
Source: PLoS One. 2011 Apr 20;6(4):e18646. doi: 10.1371/journal.pone.0018646 (PMC3080376; doi:10.1371/journal.pone.0018646)
Supplement: Supplementary Information S1 — Experimental procedures. (DOC) [file pone.0018646.s005.doc]

**Supporting Information**

**Materials and Methods**

## Cultivation of primary mouse hepatocytes

Cells were plated on rat tail collagen I-coated tissue culture dishes in William´s medium E (WME, Biochrom) supplemented with 10% FCS, 100 nM dexamethasone, 2 mM L-glutamine and 1%-penicillin/streptomycin solution (all reagents from Gibco). To allow hepatocytes to attach, cells were kept in a humidified atmosphere at 37°C and 5% CO_2_ for 4 h. Subsequently, the FCS containing WME was removed and replaced by serum-free WME supplemented with 100 nM dexamethasone, 2 mM L-glutamine and 1%-penicillin/streptomycin solution. Following incubation in this medium for 4 h, hepatocytes were washed three times and further kept in starvation medium (WME supplemented with 2 mM L-glutamine and 1%-penicillin/streptomycin solution) for 16-24 h.

**Cultivation of mouse embryonic fibroblasts**

WT 3T9 immortalized MEF cells were cultured in DMEM (4.5 g/L glucose) supplemented with 10% FCS (Biochrom) and 1% glutamine in a 5% CO_2_ incubator at 37 °C.

**Preparation of total and nuclear lysates of primary mouse hepatocytes**

For total extracts, 2 x 10^6^ cells were centrifuged at 2150 x g, 4°C for 3 min, washed with PBS, centrifuged again and lysed in 140 µl lysis buffer (20 mM Tris/HCl pH 7.4, 136 mM NaCl, 2 mM EDTA, 10% glycerol, 4 mM benzamidine, 50 mM β-glycerophosphate, 20 mM Na-diphosphate, 10 mM NaF, 1 mM Na_3_VO_4_, 1% Triton X-100 supplemented with the protease inhibitors 5 µg/ml aprotinin, 5 µg/ml leupeptin, 0.2 mM AEBSF) by shaking at 4°C for 20 min followed by a final centrifugation at 20’800 x g, 4°C for 10 min.

Nuclear extracts were prepared as described before [[1]](https://wizfolio.com/?citation=1&ver=3&ItemID=341&UserID=5577&AccessCode=4EC56EBB9A284CBF885445DB78B41BF9&CitationSuffix=). Briefly, 1 x 10^6^ cells were centrifugated (2150 x g, 4°C, 3 min), the pellet was resuspended using 400 µl buffer A (10 mM Hepes/KOH pH 7.6, 15 mM KCl, 2 mM MgCl_2_, 0.1 mM EDTA pH 8.0) and incubated on ice for 10 min. After centrifugation (2150 x g, 4°C, 3 min) buffer A was replaced by 200 µl buffer A containing 0.2% NP-40 supplemented with Complete® protease inhibitors (Roche Applied Science) and incubated for exactly 5 min on ice to lyse the cytoplasma membrane. After centrifugation (8062 x g, 4°C, 2 min) pellets were resuspended in 50 µl buffer C (25 mM Hepes/KOH pH 7.6, 50 mM KCl, 0.1 mM EDTA pH 8.0, 10% glycerol, Complete® protease inhibitors) and kept on ice. After 5 min, 4.5 µl of a 5 M NaCl solution was added and incubated for 30 min with gentle shaking at 4°C. After centrifugation (20’800 x g, 4°C, 10 min) the supernatant was isolated as nuclear extract.

**Preparation of total cell lysates of mouse embryonic fibroblasts**

For total extracts, 1 x 10^6^ cells were incubated with 100 µl lysis buffer (20mM Tris-HCL pH 7.5, 150 mM NaCL, 5 mM EDTA pH 8.0, 1 mM Na_3_VO_4_, 3 mM β-glycerophosphate, 10 mM NaF, 1 % Triton-X supplemented with the protease inhibitors aprotinin 20 µg/ml, leupeptin 20 µg/ml, pepstatin-A400 ng/ml, PMSF 200µM and proteasom inhibitor MG-132 200µM) for 10 min on ice, harvested and centrifuged at 20’800 x g, 4°C for 5 min. The supernatant was collected.

## Electrophoretic mobility shift assay (EMSA)

Nuclear protein extracts were prepared as described above. Electrophoretic mobility shift assay was performed as described before [[2]](https://wizfolio.com/?citation=1&ver=3&ItemID=341&UserID=5577&AccessCode=4EC56EBB9A284CBF885445DB78B41BF9&CitationSuffix=). Briefly, equal amounts of nuclear proteins (4 µg) were added to a reaction mixture containing 20 µg bovine serum albumin, 2 µg poly(dI-dC) (Roche Molecular Biochemicals), 2 µl buffer D+ (20 mM HEPES, pH 7.9, 20 % glycerol, 100 mM KCl, 0.5 mM EDTA, 0.25 % NP-40, 2 mM DTT, 0.1 % PMSF), 4 µl buffer F (20 % Ficoll 400, 100 mM HEPES, 300 mM KCl, 10 mM DTT, 0.1 % PMSF) and 100,000 cpm (Cerenkov) of a P33-labeled oligonucleotide for NF‑κB made up to a final volume of 20 µl with distilled water. For competition experiments (not shown) the reaction mixture contained a 100-fold excess of the respective non-radioactive labeled oligonucleotide. NF‑κB oligonucleotide (5’-AGT TGA GGG GAC TTT CCC AGG C-3’, Promega) was labeled using [γ33P]ATP (3000 Ci/mmol, Amersham Biosciences) and a T4 polynucleotide kinase (New England Biolabs). After 25 min of incubation at room temperature the samples were resolved through non-denaturing 6 % polyacrylamide gel electrophoresis and then the dried gel was exposed to an Imaging Plate (BAS-MS 2340, Fujifilm) overnight which was finally analyzed using a FLA-3000 (Fujifilm). In the figures the resulting images are shown together with the quantified 33P-stimulated luminescence (PSL) units of each specific shift. Dimer composition was determined by supershift analysis (not shown) using specific antibodies for p65 and p50 NF‑κB subunits (from Santa Cruz Biotechnologies).

## References

1. Schlatter R, Schmich K, Avalos Vizcarra I, Scheurich P, Sauter T, et al. (2009) ON/OFF and Beyond - A Boolean Model of Apoptosis. PLoS Comput Biol 5: e1000595.

2. Götschel F, Kern C, Lang S, Sparna T, Markmann C et al. **(2008)** The inhibition of GSK differentially modulates NF-κB, CREB, AP-1 and β-catenin in signaling in hepatocytes, but fails to promote TNF-α-induced apoptosis. Exper Cell Res 314: 1351-1366.
